# Cell Shape and Matrix Stiffness Impact Schwann Cell Plasticity via YAP/TAZ and Rho GTPases

**DOI:** 10.3390/ijms22094821

**Published:** 2021-05-01

**Authors:** Zhenyuan Xu, Jacob A. Orkwis, Greg M. Harris

**Affiliations:** 1 Department of Chemical and Environmental Engineering, University of Cincinnati, Cincinnati, OH 45221, USA; xuzy@mail.uc.edu (Z.X.); orkwisja@mail.uc.edu (J.A.O.); 2 Department of Biomedical Engineering, University of Cincinnati, Cincinnati, OH 45221, USA; 3 Neuroscience Graduate Program, College of Medicine, University of Cincinnati, Cincinnati, OH 45267, USA

**Keywords:** Schwann cell, mechanobiology, Rho GTPase, YAP/TAZ, extracellular matrix, peripheral nerve

## Abstract

Schwann cells (SCs) are a highly plastic cell type capable of undergoing phenotypic changes following injury or disease. SCs are able to upregulate genes associated with nerve regeneration and ultimately achieve functional recovery. During the regeneration process, the extracellular matrix (ECM) and cell morphology play a cooperative, critical role in regulating SCs, and therefore highly impact nerve regeneration outcomes. However, the roles of the ECM and mechanotransduction relating to SC phenotype are largely unknown. Here, we describe the role that matrix stiffness and cell morphology play in SC phenotype specification via known mechanotransducers YAP/TAZ and RhoA. Using engineered microenvironments to precisely control ECM stiffness, cell shape, and cell spreading, we show that ECM stiffness and SC spreading downregulated SC regenerative associated proteins by the activation of RhoA and YAP/TAZ. Additionally, cell elongation promoted a distinct SC regenerative capacity by the upregulation of Rac1/MKK7/JNK, both necessary for the ECM and morphology changes found during nerve regeneration. These results confirm the role of ECM signaling in peripheral nerve regeneration as well as provide insight to the design of future biomaterials and cellular therapies for peripheral nerve regeneration.

## 1. Introduction

Schwann cells (SCs), as the major glial cells within the peripheral nervous system (PNS), play a vital role in facilitating nerve regeneration following traumatic injury [1]. Upon injury, the injured nerve undergoes Wallerian degeneration, while the surrounding SCs dedifferentiate into a “regenerative” phenotype characterized by the upregulation of markers such as c-Jun, p75NTR, and Sox2 [2,3,4]. In addition, SCs interact and remodel the extracellular matrix (ECM) by aligning and elongating to form a cellular bridge called the “Band of Büngner”, which provides physical and trophic support for the growth of axons [5]. While forming Bands of Büngner, SCs interact with fibroblasts and activate ephrin-B/EphB2 signaling for guided directional migration of the SCs and to expedite axon growth [6]. Despite the intrinsic regenerative potential possessed by SCs, spontaneous functional recoveries of PNS injuries are often very poor in the cases of severe traumatic injuries. This is due to a decrease in the SC regenerative phenotype resulting from long-term denervation of SCs within nerve stumps [7,8]

During the nerve regeneration period, SCs maintain direct contact with the ECM. A reciprocity exists between matrix deposition and cell function by way of cues such as the ECM itself [9,10,11]. It has also recently been established that ECM stiffness regulates SC behaviors including proliferation, migration, and the expression of regenerative markers [12,13,14]. Differences in the ECM between healthy and regenerating nerves are abundant, including increased fibronectin and laminin content during demyelination and remyelination phases of nerve regeneration [15,16]. Cell morphology is also known to be regulated by the ECM and has been shown to play an important role in modulating SC behaviors. For instance, our lab has recently demonstrated that cell spreading inhibition and cell elongation upregulates c-Jun expression, a key regenerative marker [14,17]. However, many specific mechanisms regulating SC mechanobiology remain unclear [10].

Focal adhesions (FAs) and the actin cytoskeleton are integral components in the mechanotransduction process, where ECM influence is interpreted by transducing extracellular cues through the actin cytoskeleton to generate biological responses [18]. FAs regulate important transcriptional factors involved in mechanobiology such as YAP (Yes-associated protein)/TAZ (PDZ-binding motif). As the ECM increases in stiffness, or the cell spreading area becomes larger, FAs activate YAP/TAZ via FAK (focal adhesion kinase)/tyrosine kinase SRC, which is characterized by the nuclear translocation of YAP/TAZ [19,20,21]. YAP/TAZ also promotes SC myelination via Krox-20 signaling during nerve development and initiates remyelination during nerve regeneration, whereas YAP/TAZ inactivation results in a complete failure to remyelinate regenerated axons [22,23,24,25,26,27]. In conjunction with the role of FAs, the actin cytoskeleton comprises a vital component of mechanotransduction, where the remodeling of cytoskeletal structure balances external stimuli exerted by the ECM [18]. Remodeling of the actin cytoskeleton particularly affects the expression of Rho GTPases, a group of small G proteins comprising RhoA and Rac1, among others, where the activation of Rho GTPases can be characterized by their binding with guanosine triphosphate (GTP). Cells with larger spreading area or stiffer ECM can stimulate stress fiber formation by upregulating RhoA activity [28,29]. Rac1 was found to induce the assembly of membrane protrusions such as lamellipodium, which can promote cell migration, polarization, and elongation [30,31,32]. RhoA and Rac1 have both been implicated in multiple SC functions with unclear definitive mechanisms. For example, RhoA activity was found to be indispensable for initiation of the myelination program in early stages, while RhoA has also been shown to be responsible for activating the JNK/c-Jun signaling that upregulates non-myelinating SC markers [33,34]. Rac1 is upstream of the MAPK kinase 7 (MKK7)/JNK/c-Jun signal cascade, and thus would seemingly promote SC demyelination. However, myelination in mice SCs is attenuated upon Rac1 activity inhibition [35,36,37].

Herein, we deconstruct the role of ECM stiffness and cell shape involved in the SC phenotype by investigating the roles of Rho GTPases and YAP/TAZ. We have generated an engineered micropatterned polydimethylsiloxane (PDMS) cell culture platform in order to individually tune mechanical ECM conditions such as stiffness and cell adhesive area. Changes in the mechanotransducer activity for SCs by different conditions were examined by microarray analysis, immunofluorescence staining, and Western blot. To further analyze influences of the ECM on SC phenotypes, transcriptional inhibitors and small interfering RNA (siRNA) were employed, and the SC regenerative capacity was quantified by the expression of SC markers such as c-Jun, p75NTR and Sox-2. We conclude that an ECM with a high Young’s modulus and well-spread cells inhibits key SC regenerative markers by the activation of RhoA and YAP/TAZ signaling, and that higher degrees of cellular elongation promote key regenerative markers by the activation of Rac1/MKK7/JNK signaling. Altogether, our data illustrate the importance of the ECM microenvironment as a regulator for SC phenotypes by Rho GTPases and YAP/TAZ.

## 2. Results

### 2.1. Matrix Stiffness Inhibits c-Jun and p75NTR with Activation of RhoA and YAP/TAZ

**Figure 1:**
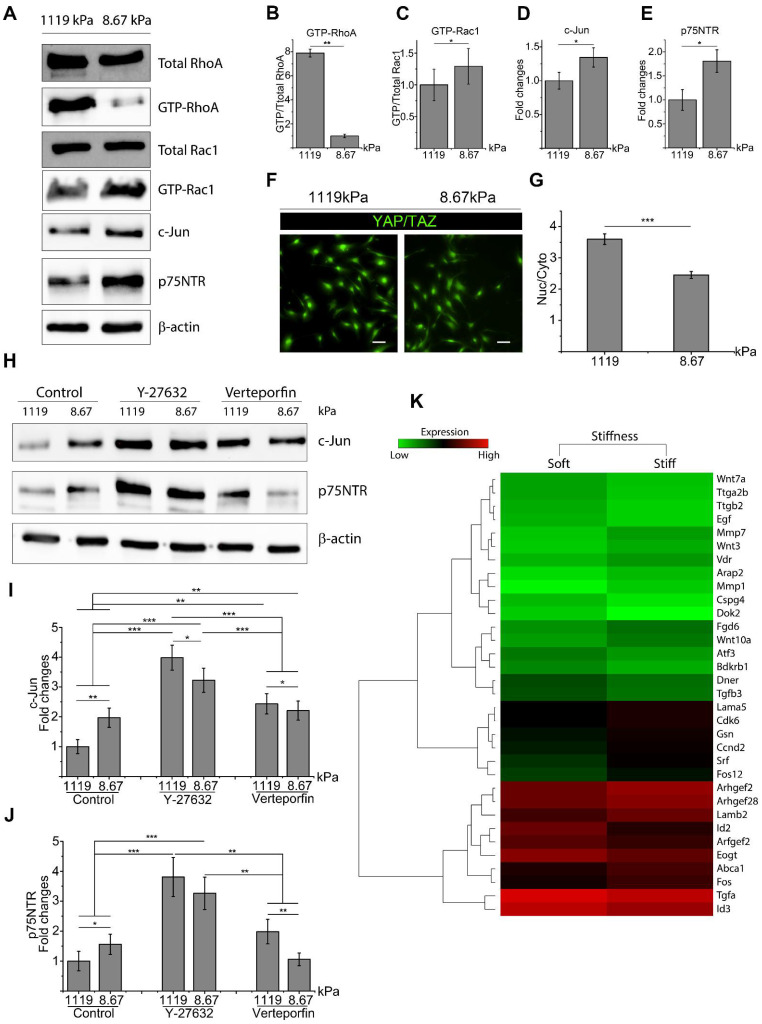
Increases in extracellular matrix (ECM) stiffness promoted RhoA and YAP/TAZ activity, which led to decreased expression of regenerative markers. (**A**–**E**) Western blot shows that the expression of key factors can be regulated by ECM stiffness in Schwann cells (SCs). Four unique cell lysates were created for Western blots. (**F**,**G**) Nuclear YAP/TAZ (green) is upregulated as ECM stiffness increases. A total of 98 cells were quantified per well, with 6 wells quantified in each of 2 unique trials. Scale bar = 50 μm. (**H**–**J**) Expression of c-Jun and p75NTR are enhanced by RhoA and YAP/TAZ inhibition, with RhoA inhibition resulting in higher expression compared to YAP/TAZ inhibition. Three lysates for c-Jun and two lysates for p75NTR were created for Western blots. (**K**) Heatmap generated by microarray analysis shows up- and downregulated genes that are associated with either mechanotransduction or SC regenerative phenotypes. Data are presented as the mean ± SEM. * *p* < 0.05, ** *p* < 0.005, *** *p* < 0.0005, *p*-values found in Supplemental Table S1.

To analyze the interplay between the ECM and SC phenotype, SCs were seeded on laminin-coated PDMS substrates with a variable Young’s modulus (E) of either 1119 ± 72.26 kPa (stiff) or 8.67 ± 0.48 kPa (soft). It was found that on a stiff substrate, RhoA activity is significantly upregulated in SCs (Figure 1A,B). However, when analyzing Rac1 expression, there was no significant difference in total Rac1 expression found for SCs seeded on a stiff versus soft substrate. However, active Rac1 was found to be downregulated in SCs seeded on the stiff ECM, which is consistent with an antagonistic relationship between active RhoA and Rac1 for maintaining cytoskeletal stability (Figure 1A,C) [38]. The key mechanotransducer YAP/TAZ was also examined by immunofluorescence staining and quantified by the ratio of nuclear to cytoplasmic fluorescent intensity. These results showed that a stiff substrate significantly enhanced the activity of YAP/TAZ compared to a soft substrate (Figure 1F,G). Conversely, expressions of key regenerative markers c-Jun and p75NTR were downregulated on the stiff ECM (Figure 1A,D,E).

### 2.2. RhoA and YAP/TAZ Inhibition Promote SC Regenerative Markers

To begin to decouple the relationship of ECM effectors on RhoA and YAP/TAZ expression, SCs were treated with either Y-27632 to inhibit Rho-associated protein kinase activity or Verteporfin to inhibit YAP/TAZ activity. Rho-associated protein kinase is a downstream effector of RhoA; therefore, treatment with ROCK inhibitor Y-27632 is commonly utilized to inhibit RhoA signaling [39]. Following Y-27632 treatment, active RhoA was significantly downregulated while Rac1 activity was upregulated (Figure S1C–E). Y-27632 treatment also resulted in a decrease of active YAP/TAZ, which was characterized by the translocation of YAP/TAZ to the cytoplasmic area (Figure S1A,B). Verteporfin treatment also significantly downregulated YAP/TAZ and RhoA activity, but to a lesser extent when compared to Y-27632 treatment (Figure S1). Western blot analysis was performed to identify the expression of SC regenerative markers following RhoA or YAP/TAZ inhibition. After Y-27632 treatment, both c-Jun and p75NTR expression were significantly upregulated for SCs seeded on both stiff and soft substrates compared to untreated SCs (Figure 1H). Interestingly, both Y-27632 and Verteporfin treatment resulted in the opposite response between key markers and ECM stiffness, where a stiff ECM promoted increased expression of c-Jun and p75NTR following inhibition (Figure 1H–J). Furthermore, only c-Jun was significantly upregulated following Verteporfin treatment, whereas no significant change was seen in p75NTR expression (Figure 1H,J). Inhibition of active RhoA and YAP/TAZ by Y-27632 and verteporfin significantly promoted SC regenerative phenotype but to different extents, with RhoA inhibition resulting in higher SC regenerative potential compared to YAP/TAZ inhibition. Furthermore, according to microarray data (Figure 1K), critical transcriptional factors and proteins such as *Arfgef2*, *Arhgef2*, *Srf*, *Fos*, *Cdk6*, *Bdkrb1*, *Gsn*, *Abca1*, which are reported to be related to RhoA/YAP/TAZ signaling, were upregulated for SCs seeded on stiff substrates [40,41,42,43,44,45]. Meanwhile, SC regenerative genes were upregulated for SCs seeded on soft substrates, such as *EGF*, *Atf3*, *Cspg4 (Ng2)*, *Id2*, *Mmp7*, and Wnt signaling [46,47,48,49].

### 2.3. Rac1 Cooperatively Modulates SC Phenotype with RhoA and YAP/TAZ

**Figure 2:**
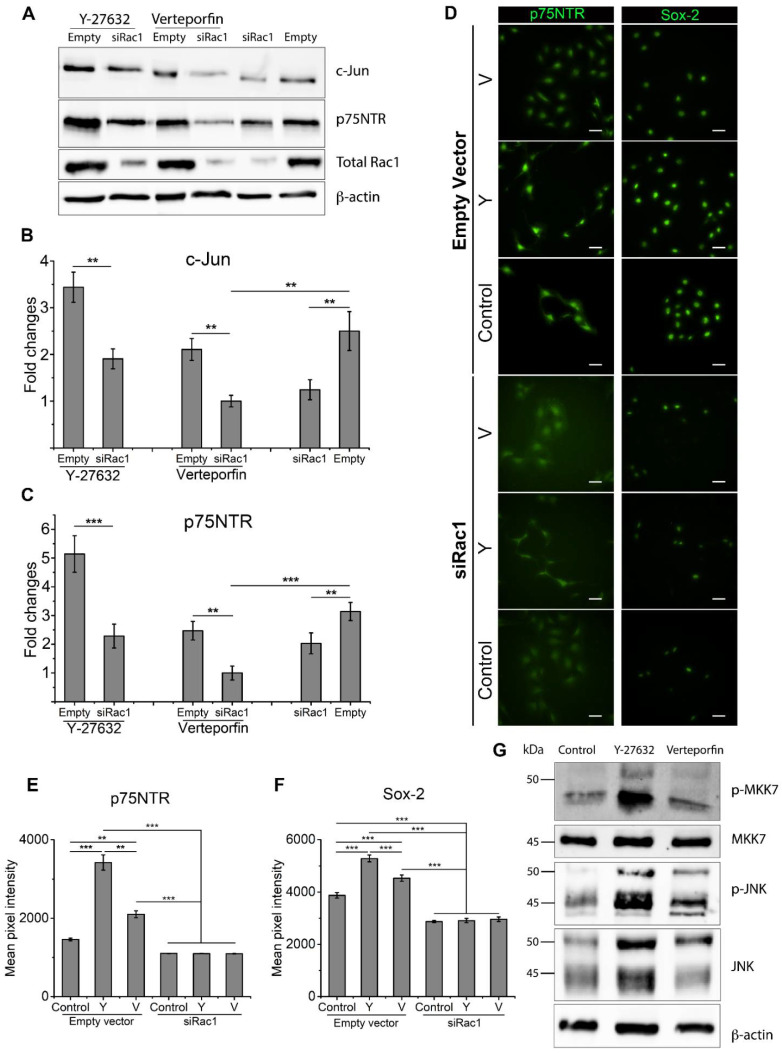
RhoA and YAP/TAZ inhibition led to increased expression of regenerative markers c-Jun, p75NTR, and Sox-2 through the activation of Rac1/MKK7/JNK signaling. (**A**–**C**) Active Rac1 inhibition via siRac1 significantly lowers c-Jun and p75NTR expression with RhoA and YAP/TAZ inhibition, showing no change in expression when Rac1 activity is blocked by siRac1 transfection. Four cell lysates were created for Western blot quantification. (**D**–**F**) Immunofluorescent stained Schwann cells treated with a combination of Y-27632, Verteporfin, or siRac1 showing expression of p75NTR and Sox-2. Three unique trials with a minimum of 42 cells per trial were quantified. Scale bar = 50 μm. (**G**) Rac1/MKK7/JNK expression when treated with Y-27632 or Verteporfin. Data are presented as the mean ± SEM. ** *p* < 0.005, *** *p* < 0.0005, *p*-values found in Supplemental Table S2.

It has been demonstrated that RhoA inhibition resulted in the activation of Rac1 and promotion of SC regenerative markers. To elucidate the role of Rac1 in SC phenotype, SCs were transfected with Rac1 siRNA (siRac1). Rac1 inhibition was confirmed by Western blot following siRac1 transfection (Figure S2). c-Jun and p75NTR were also significantly downregulated following Rac1 inhibition, consistent with our previous results showing that Rac1 activation led to a higher SC regenerative capacity (Figure 2A). For the confirmation of Rac1 involvement in RhoA and YAP/TAZ inhibition, Y-27632 and Verteporfin-treated SCs were transfected with siRac1 or an empty vector. Figure 2A–C show that c-Jun and p75NTR expression levels were significantly downregulated following Rac1 inhibition when compared to SCs transfected with empty vectors. For Y-27632-treated SCs, expressions of both c-Jun and p75NTR were downregulated to a level similar to untreated SCs following siRac1 transfection (Figure 2A–C). The downregulation of SC markers caused by siRac1 transfection was higher for Verteporfin-treated SCs, where expression levels of both c-Jun and p75NTR were significantly lower when compared to untreated SCs (Figure 2A–C).

Expression levels of p75NTR and Sox-2, as measured by average fluorescent intensity from immunofluorescent staining, showed consistent results (Figure 2D–F). For SCs transfected with empty vectors, RhoA and YAP/TAZ inhibition significantly enhanced the fluorescent intensity of both p75NTR and Sox-2. For SCs transfected with siRac1, the expression of p75NTR decreased to a level comparable to untreated SCs (Figure 2E), and the expression of Sox-2 was significantly lower than in untreated SCs (Figure 2F). To further establish that Rac1 is activated following RhoA and YAP/TAZ inhibition, Western blot analysis was performed for RhoA- and YAP/TAZ-inhibited SCs. Western blot showed that levels of phosphorylated-MKK7, phosphorylated-JNK, and total JNK were upregulated upon either Y-27632 or Verteporfin treatment, with RhoA inhibition resulting in higher activity of Rac1/MKK7/JNK signaling compared to YAP/TAZ inhibition (Figure 2G). These results remain consistent with previous findings that RhoA inhibition resulted in higher expression levels of key SC regenerative markers than YAP/TAZ inhibition. In turn, RhoA and YAP/TAZ inhibition activated Rac1/MKK7/JNK signaling to promote SC regenerative markers.

### 2.4. Cell Morphology Impacts RhoA, Rac1, and YAP/TAZ to Determine SC Phenotype

**Figure 3:**
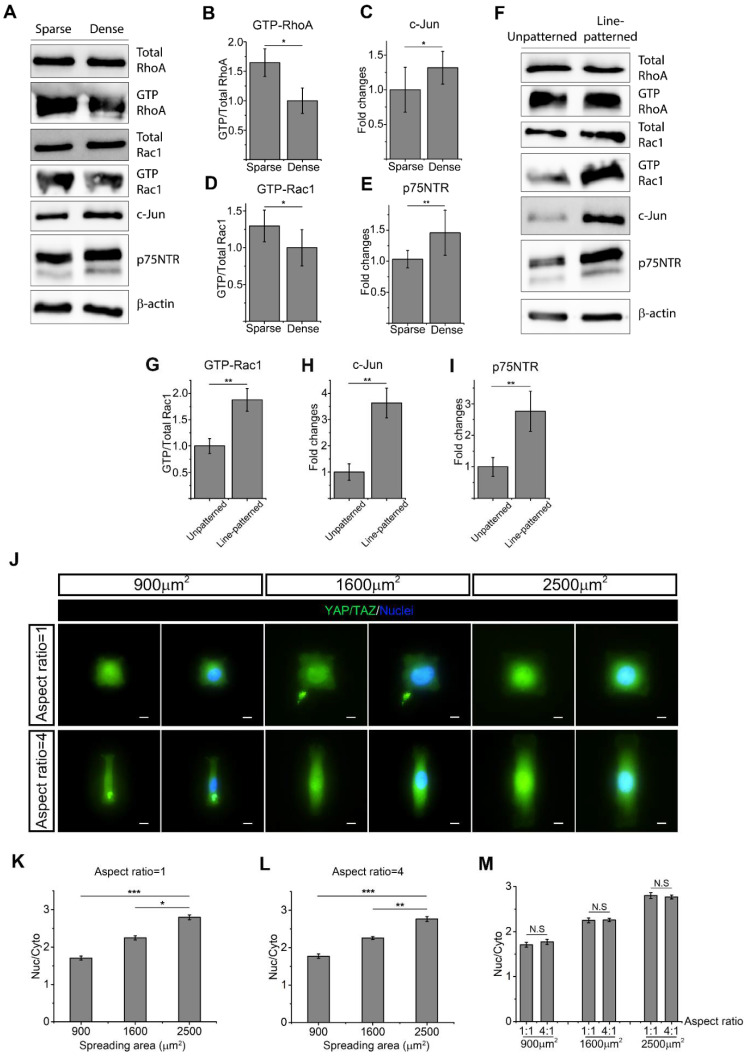
Schwann cell (SC) morphology regulates the expression of active RhoA, Rac1 and YAP/TAZ. (**A**–**E**) Western blot quantification of SCs show RhoA, Rac1, c-Jun, and p75NTR expression of different cell spreading areas. Four cell lysates were created for Western blot quantification. (**F**–**I**) Western blot quantification of SCs shows RhoA, Rac1, c-Jun, and p75NTR expression of different extents of cell elongation. Three cell lysates were created for Western blot quantification. (**J**) Immunofluorescent staining of micropatterned SCs shows nuclear (active) YAP/TAZ (green) with different spreading areas and extents of elongation. Scale bar = 10 μm. (**K**,**L**) When the extent of cell elongation is constant, active YAP/TAZ declines as cell spreading area decreases. (**M**) When SCs process the same spreading area, active YAP/TAZ cannot be regulated by the extent of cell elongation. For YAP/TAZ quantification of micropatterned SCs, 3 unique trials with a minimum of 89 single micropatterned SCs per trial were quantified. Data are presented as the mean ± SEM. * *p* < 0.05, ** *p* < 0.005, *** *p* < 0.0005, *p*-values found in Supplemental Table S3.

SC morphology, including total spreading area and elongation, has been linked with ECM stiffness. To explore the roles of morphology and ECM stiffness in SC phenotypes, we first controlled the average SC spreading area by initial culture density. As expected, it was shown that a higher seeding density significantly reduced the average spreading area (Figure S3A,B). Western blot analysis demonstrated there was no significant difference in total RhoA and total Rac1 expression for SCs with different average spreading areas (Figure 3A). However, active RhoA and Rac1 were significantly upregulated for SCs that possessed a larger average spreading area (Figure 3A,B,D). For SC markers, both c-Jun and p75NTR were downregulated in SCs with a larger spreading area (Figure 3A,C,E). Finally, immunofluorescence staining showed YAP/TAZ activity on micropatterned SCs of different spreading area and elongation, with cell morphology precisely controlled and cell–cell contact eliminated (Figure 3J). Nuclear YAP/TAZ quantification showed that YAP/TAZ translocated into the nuclear area as SC spreading area became larger, indicating an increased YAP/TAZ activity (Figure 3K,L). As reported in Section 2.3, active Rac1 upregulated certain SC markers, suggesting that cell spreading directs the SC phenotype via RhoA signaling, rather than Rac1 signaling.

Elongation of SCs is crucial to the formation of the Bands of Büngner that promote nerve regeneration post injury [50]. SC elongation was also assessed by utilizing line-patterned substrates and unpatterned substrates, with line-patterned substrates significantly enhancing SC elongation (Figure S3C,D). Western blot analysis shows that Rac1 activity was significantly higher in elongated SCs (Figure 3F,G). By contrast, a different extent of elongation did not result in significant changes in total RhoA expression, but elongated SCs slightly increased active RhoA. Elongated SCs also exhibited a higher expression of both c-Jun and p75NTR (Figure 3H,I). Nuclear YAP/TAZ was also quantified in micropatterned SCs of the same spreading area, but with different extents of elongation. It was found that SCs with a different extent of elongation exhibited similar levels of nuclear YAP/TAZ (Figure 3M). Taken together, SC elongation upregulated Rac1 activity and SC regenerative markers without a significant change in the expression of active RhoA and YAP/TAZ.

### 2.5. SC Spreading Promotes Active RhoA and YAP/TAZ and Inhibits c-Jun and p75NTR

**Figure 4:**
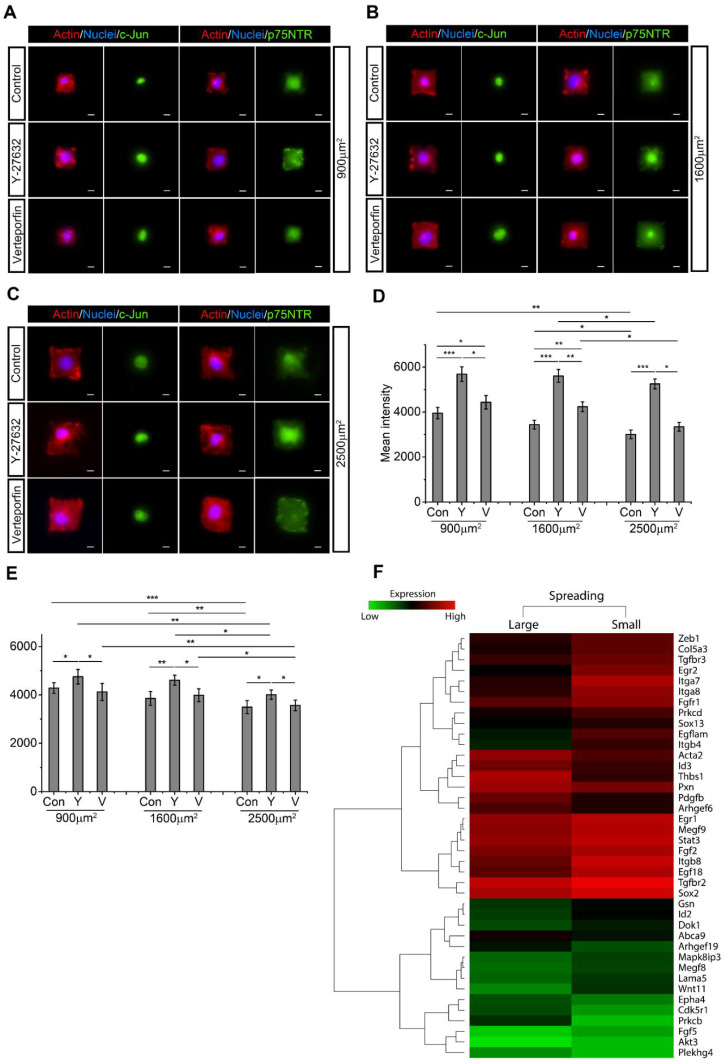
When cell spreading area remains constant, inhibition of RhoA and YAP/TAZ significantly upregulate the expression of c-Jun and p75NTR. Schwann cells (SCs) were seeded into (**A**) 900 μm^2^, (**B**) 1600 μm^2^, or (**C**) 2500 μm^2^ micropatterned cell adhesive islands. F-actin (red), nucleus (blue), c-Jun (green), and p75NTR (green) are visualized by immunofluorescence staining. Scale bar = 10 μm. (**D**,**E**) Statistical analysis shows the expression of c-Jun and p75NTR after Y-27632 and Verteporfin treatment. Mean pixel fluorescent intensities are measured for quantification. For fluorescent intensity quantification of micropatterned SCs, 5 unique trials with a minimum of 51 single micropatterned SCs per trial were quantified. (**F**) Microarray analysis indicates that SC with larger spreading area showed higher RhoA- and YAP/TAZ-associated genes and a decline in SC regenerative markers. Data are presented as the mean ± SEM. * *p* < 0.05, ** *p* < 0.005, *** *p* < 0.0005, *p*-values found in Supplemental Table S4.

To examine the impact of SC spreading on RhoA and YAP/TAZ activity, micropatterned SCs of different spreading area were treated with either RhoA or YAP/TAZ inhibitors, and c-Jun and p75NTR expressions were quantified by fluorescent intensity (Figure 4A–C). Without inhibitor treatment, both c-Jun and p75NTR expression were downregulated as cell spreading increased from 900 µm^2^ to 2500 µm^2^ (Figure 4D,E). Both RhoA and YAP/TAZ inhibition significantly upregulated c-Jun expression when cell spreading area was maintained, with RhoA inhibition resulting in a higher c-Jun expression compared to YAP/TAZ inhibition (Figure 4D). Interestingly, following RhoA inhibition, there was no significant change in c-Jun expression level for SCs of different spreading areas. However, when YAP/TAZ was inhibited, SCs with the largest spreading area (2500 µm^2^) still resulted in the lowest expression of c-Jun. Additionally, only RhoA inhibition promoted p75NTR expression, whereas YAP/TAZ inhibition did not significantly increase p75NTR expression (Figure 4E). Furthermore, despite the inhibition of RhoA and YAP/TAZ, p75NTR expression decreased as cell spreading area increased, suggesting that cell spreading area regulates p75NTR expression independent of RhoA and YAP/TAZ (Figure 4E). Microarray analysis further confirmed that SC spreading promoted activity of RhoA/YAP/TAZ-associated genes, such as *Arhgef6*, *Arhgef19*, *Pxn*, *Epha4*, *Acta2* (Figure 4F) [51,52,53,54]. However, SC regenerative markers such as *Sox2*, *Id2*, Wnt signaling, *Egr1*, Egf-like domain, *Fgf*, *Mapk8ip3* and *Stat3* were upregulated for SCs with a small spreading area [46,55,56].

### 2.6. Active Rac1 Promotes SC Regenerative Markers through SC Elongation

**Figure 5:**
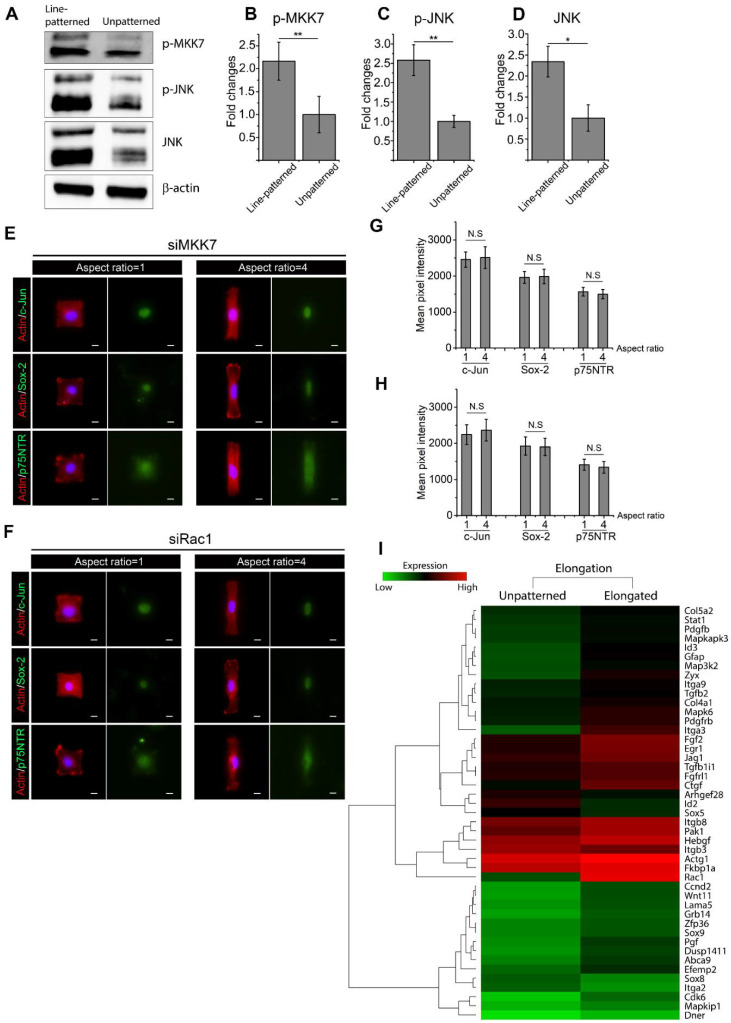
Schwann cell (SC) elongation promotes a regenerative phenotype via the Rac1/MKK7/JNK signal cascade. (**A**–**D**) Rac1/MKK7/JNK signaling is activated when SCs are seeded on line-patterned substrates compared to unpatterned surface. Two cell lysates were created for Western blot quantification. (**E**,**F**) SCs were transfected with MKK7 and Rac1 siRNA and subsequently seeded on micropatterned cell adhesive islands where cell spreading area was maintained at 1600 μm^2^ but with different extents of elongation. Immunofluorescence staining was performed to stain F-actin (red), nucleus (blue), c-Jun (green), Sox-2 (green) and p75NTR (green). Mean pixel intensities of c-Jun, Sox-2 and p75NTR were measured to quantify SC regenerative capacity. For fluorescent intensity quantification of micropatterned SCs, 4 unique trials with a minimum of 30 single micropatterned SCs per trial were quantified. Scale bar = 10 μm. (**G**,**H**) Statistical analysis shows that cell elongation is not able to promote SC regenerative phenotypes after Rac1/MKK7/JNK signaling inhibition. (**I**) Microarray analysis shows activation of Rac1-associated signaling and SC regenerative markers within elongated cells. Data are presented as the mean ± SEM. * *p* < 0.05, ** *p* < 0.005, *p*-values found in Supplemental Table S5.

To further explore the role of cellular elongation undertaken by SCs following injury, Western blot analysis targeting Rac1/MKK7/JNK was performed on either line-patterned substrates or unpatterned substrates. Upregulation in phosphorylated-MKK7, phosphorylated-JNK, and total JNK was observed in line-patterned SCs (Figure 5A–D), suggesting the activation of Rac1/MKK7/JNK in elongated SCs. To confirm the role of active RhoA, SCs were micropatterned into cell adhesive islands using differing extents of elongation before treatment with RhoA or YAP/TAZ inhibitors. Elongated SCs still exhibited increased levels of SC regenerative markers following RhoA and YAP/TAZ inhibition, suggesting that elongation did not require RhoA activity to promote SC markers (Figure S4).

Rac1/MKK7/JNK signaling was inhibited by transfecting SCs with Rac1 siRNA (siRac1) and MKK7 siRNA (siMKK7). Inhibition was verified by Western blot, where siMKK7 and siRac1 transfection resulted in a significant downregulation of Rac1/MKK7/JNK activity (Figure S5). siMKK7- and siRac1-transfected SCs were then micropatterned into cell adhesive islands of equal area, but differing aspect ratios for the quantification of Sox-2, c-Jun, and p75NTR by fluorescent intensity (Figure 5E,F). SC elongation did not result in significant changes in the expression levels of any of c-Jun, Sox-2, or p75NTR, indicating a significant role for Rac1/MKK7/JNK signaling in SC morphology changes (Figure 5G,H). Furthermore, microarray analysis indicated that Rac1/JNK signaling was activated for elongated SCs, which was characterized by the upregulation of *Zyx*, *Rac1*, and *Pak1* (Figure 5I) [57,58]. SC elongation also resulted in the upregulation of SC regenerative markers Egf-like domain, MAPK signaling, *Id2*, Wnt signaling, *Fgf* and *Egr1* [46].

## 3. Discussion

**Figure 6:**
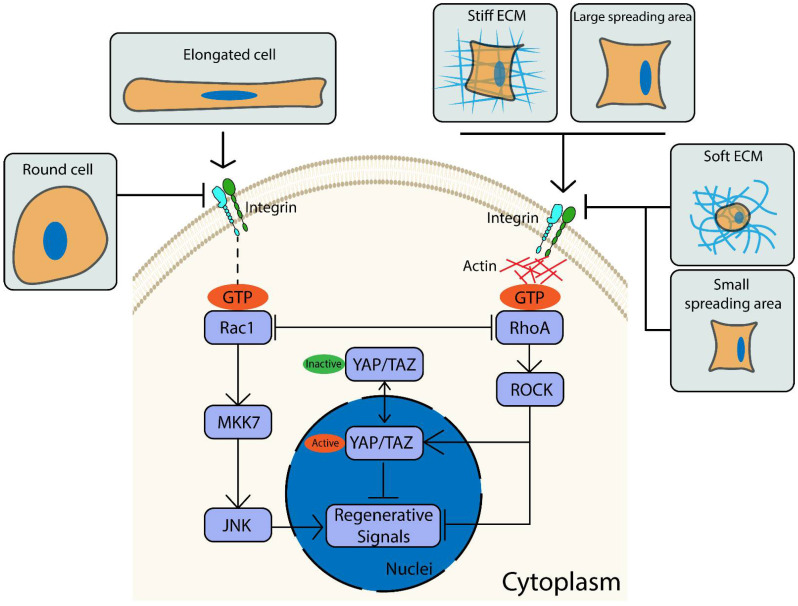
Extracellular matrix (ECM) properties are essential to regulate Schwann Cell (SC) phenotypes. The proposed mechanism shows how cell spreading, ECM stiffness, and key proteins influence the SC phenotype.

Here, we show that the complex interplay between the SC phenotype and the ECM is mediated, at least in part, by Rho GTPase and YAP/TAZ signaling (Figure 6). Specifically, ECM stiffness and cell spreading block key SC regenerative markers by upregulating active RhoA and YAP/TAZ. Furthermore, blocking RhoA and YAP/TAZ leads to increased expression of regenerative markers on a stiff ECM and larger spreading areas by the activation of Rac1/MKK7/JNK signaling. Rac1 signaling also plays a vital role in regulating the SC phenotype through cellular elongation, where SC elongation showed no change in the activities of both RhoA and YAP/TAZ. Inhibition of Rac1 significantly suppressed the expression of SC regenerative markers in elongated SCs to a level similar to non-elongated SCs, further confirming the role of Rac1 in SC shape.

Cell–ECM interactions are vital to cellular function, and many ECM cues such as stiffness have previously been reported to regulate SC morphology and proliferation [14,59,60,61]. ECM properties continuously fluctuate during injury and in disease states. Therefore, fully elucidating the signaling relationships between SCs and the ECM is critical for the next generation of PNS therapies. Furthermore, mechanical properties, such as local tissue stiffness within the PNS, are heterogeneous and can be altered by maturation and diseases such as fibrosis, emphasizing the complexity in precise measurement and the modulation of mechanical properties within the vicinity of injured sites [62,63]. However, it is challenging to acquire certain mechanical conditions that favor all cellular components within the PNS to promote nerve regeneration. For example, stiff substrates suppress SC regenerative marker expression and proliferation, although promote fibroblast spreading, proliferation, migration rate and persistence, which benefits fibroblast infiltration during the demyelination stage of nerve regeneration [64,65,66]. Furthermore, incorporating specific mechanical cues that promote SC regenerative phenotype into biomaterial designs can be burdensome, if not tunable, on a temporal scale. For example, the materials utilized to construct nerve guidance conduits (NGCs) must retain a certain stiffness in the perspective of structural rigidity and transplanting a soft NGC to the injury site may encounter problems such as collapse or swelling [67].

This study has shown that well-spread SCs on a stiff ECM exhibited higher activity of RhoA and YAP/TAZ. This was consistent with work in other cell types, which report that a stiff ECM and high degree of cell spreading induce higher cellular tension, activate FAKs, and subsequently modulate the actin cytoskeleton to upregulate mechanotransducers such as RhoA and YAP/TAZ [68]. We also showed that active Rac1 is upregulated in SCs on soft substrates, which was likewise consistent with reports that Rac1 is activated in neuronal cells seeded on soft substrates [69]. Furthermore, this work reveals a relationship between the activity of known mechanotransducers and SC plasticity, providing insights into the intracellular signaling involved in SC sensing of the ECM. As the impact of SC plasticity is becoming more well known, it is imperative to identify molecular mechanisms relating SCs to the ECM underneath. Toward this, when RhoA and YAP/TAZ are inhibited, SCs show an increased expression of regenerative markers on stiff ECM or with larger spreading areas. Interestingly, upon YAP/TAZ inhibition, SCs on stiff substrates show higher expression of regenerative markers than SCs seeded on soft substrates. Potentially, RhoA/JNK/c-Jun signaling is activated due to the inhibition in RhoA/YAP activity. Therefore, stiff substrates that promote a higher activity of RhoA also resulted in higher expression of regenerative markers. When RhoA is inhibited by Y-27632, we show that active Rac1 was significantly upregulated. It is also found that there is no significant difference in the expression of regenerative markers for SCs on substrates of different stiffness when RhoA activity is inhibited. These results indicate that the activity of YAP/TAZ is required for substrate stiffness to regulate active Rac1, which is consistent with previous work that illustrated the inherent connection between Rac1 and YAP/TAZ [58,70]. Conversely, looking at cell shape, our results show that despite RhoA inhibition, SCs with the largest spreading area (2500 µm^2^) still exhibited the lowest activity of c-Jun and p75NTR. SCs lose cell–cell contact due to the confinement in single cell adhesive islands, thereby inhibiting the Hippo pathway. Inhibition on the Hippo pathway upregulates active YAP/TAZ by inhibiting phosphorylation and cytoplasmic translocation that may compromise YAP/TAZ inhibition by Verteporfin [71]. Therefore, it is conceivable that following RhoA and YAP/TAZ inhibition, micropatterned SCs generally exhibit higher levels of active YAP/TAZ compared to unpatterned SCs to maintain the activity of RhoA/YAP/TAZ signaling, enabling SCs to sense mechanical cues and alter SC phenotypes.

In addition to the key protein markers signifying SC plasticity, other aspects of SC function such as proliferation, migration, and cell shape must be analyzed when looking at the context of SCs in PNS regeneration [72]. With regard to YAP/TAZ, many previous in vitro studies, including one showing human epithelial cells exposed to mechanical stimuli, such as stretching, have shown higher proliferation rates due to the activation of YAP/TAZ [73]. Additionally, an in vivo study confirmed that both YAP and TAZ are required for SC proliferation, with TAZ functioning to downregulate *Gnas*, a transcriptional factor that impedes proliferation [74]. However, it is also reported that active YAP/TAZ is not required for injury-induced SC proliferation in the early phase of nerve regeneration [24]. Furthermore, Y-27632 treatment does not alter SC proliferation rates because RhoA regulates SC proliferation through AKT rather than ROCK signaling [75]. When compared to our results, it is promising to transiently suppress ROCK or YAP/TAZ activity at the onset of nerve regeneration, which may help promote SC regenerative ability without impacting SC proliferation rates. SC migration and cell shape are also critical to nerve regeneration, where SCs are responsible for forming regenerative tracks within basal lamina to promote axon survival and growth. Regarding cell shape, RhoA inhibition by Y-27632 or p190RhoGAP has been shown to hinder cell adhesion although promote cell elongation and directional migration, which is vital to nerve regeneration [33,76,77,78]. RhoA is also responsible for cell membrane retraction, which is essentially the antithesis of what is required in nerve regeneration [30]. However, another study illustrated a negative correlation between active YAP/TAZ and SC proliferation and migration by applying electromagnetic fields (EMFs). This showed that EMF-induced proliferation and migration activate the Hippo pathway, downregulating active YAP/TAZ [79]. In the context of our findings, RhoA and YAP/TAZ inhibition not only upregulated the expression of c-Jun, p75NTR and Sox-2, but also potentially enhanced other key features found in SC dedifferentiation.

Rac1, another member of the Rho GTPase family, transduces mechanical cues and regulates SC function during development and nerve regeneration [36,80]. Here, we show that cell shape changes by cellular elongation promote SC expression of c-Jun and p75NTR by activating Rac1/MKK7/JNK rather than through RhoA and YAP/TAZ signaling. This is consistent with reports that Rac1 activation promotes cell elongation and Rac1 inhibition produces rounded morphology without an impact on active YAP/TAZ [81,82]. Other markers, such as neurofibromin 2 (NF2)/merlin, have also been reported to promote SC elongation, with its downregulation resulting in a morphological transformation of SCs from bipolar to multipolar; however, NF2 overexpression downregulates SC markers such as c-Jun and p75NTR, and myelinated SC markers were downregulated in merlin-null SCs [83,84,85]. These results are not consistent with our observations, suggesting that NF2/merlin may not be involved in upregulating the SC regenerative phenotype by elongation or that further study may be necessary. We have also showed that siRac1-transfected SCs show a significant downregulation in p75NTR and Sox-2, which highlights the necessity of Rac1 expression in the function of SCs post injury. Additionally, neuregulin-1 and neurotrophin-3 promote SC migration by activating Rac1 and downstream JNK, suggesting a positive correlation between Rac1 activity and the previously discussed SC migration rate [86,87]. In terms of proliferation, Rac1 has been reported to promote SC proliferation by suppressing NF2; however, this leads to tumor formation, which often results from an unsuccessful nerve regeneration [88]. The formation of tumorigenesis cells occurs when Hippo is inhibited while YAP/TAZ is activated, leading to a loss of contact inhibition and uncontrolled proliferation [89]. Here, we observed that SC regenerative markers can be enhanced through RhoA and YAP/TAZ inhibition. Therefore, it is of interest to determine if Rac1-induced tumor formation can be avoided with RhoA or YAP/TAZ inhibition. In this way, the SC proliferation rate can be promoted by Rac1 activation, and SC regenerative markers can be upregulated by RhoA and YAP/TAZ inhibition.

## 4. Materials and Methods

### 4.1. Cell Culture

RT4-D6P2T Schwann cells (ATCC, CRL-2786) were cultured with Eagle’s minimal essential medium (DMEM), supplemented with 10% fetal bovine serum and 1% pen/strep, and incubated at 37 °C with 5% CO_2_ for cell growth and maintenance. For cell passaging, cell monolayers with 70% to 80% confluency were incubated with 0.25% trypsin in versene for 3 min to resuspend SCs for further analysis. For experimental cell density, 5000 cells/cm^2^ were cultured on line-patterned substrates, 10,000 cells/cm^2^ on unpatterned substrates, 1000 cells/cm^2^ on rectangular-patterned substrates, and 20,000 cells/cm^2^ for lysate preparation. Y-27632 (Tocris, 1254) (20 μM) was used to inhibit Rho-associated, coiled-coil-containing protein kinase (ROCK) activity, and Verteporfin (Tocris, 5305) (1 μM) was used to inhibit active (nuclear) YAP/TAZ. SCs were incubated with inhibitors overnight before proceeding with experiments.

### 4.2. Preparation of Engineered Substrates

To create cell culture substrates of differing Young’s modulus, PDMS base and curing agent (Dow) were mixed vigorously using a pipette tip at 10:1 and 50:1 ratios before degassing with vacuum desiccation. The PDMS precursor mixture was coated on glass coverslips or Petri dishes using a spin coater (Laurell Technologies, North Wales, PA, USA, WS-400-6NPP) and placed in an oven at 60 °C overnight to cure. The Young’s modulus values (E) of PDMS with mixing ratios of 10:1 and 50:1 were 1119 ± 72.26 kPa and 8.67 ± 0.48 kPa, respectively; this was retested periodically using a compressive force test machine (TestResources) [14]. PDMS values were chosen to cover the stiffness range of sciatic nerves, which varies between 5 and 50 kPa with aging and disease, and to provide adequate contrast of stiffness to study SC behaviors [21,90]. To coat laminin on PDMS substrates, PDMS surfaces were first treated with a UV–Ozone cleaner (Novascan, Ames, IA, USA) for 7 min before incubating with 10 µg/mL laminin (Thermo Fisher, Waltham, MA, USA, 23017015) solution at 37 °C for 30 min.

To control cell shape and area by micropatterning, PDMS stamps with square/rectangular or line patterns were prepared by standard photolithography using photoresist SU-8 2010 (Microchem Corp, Newton, MA, USA), which is detailed elsewhere [14,91]. The patterned side of the PDMS stamp was incubated with 50 µg/mL laminin solution for 1 h before bringing it into conformal contact with the cell culture substrate for 5 min. After printing, PDMS stamps were removed and the cell culture substrates were incubated with 0.2% *w*/*v* Pluronic F-127 (Sigma-Aldrich, St. Louis, MO, USA, P2443) solution for 1 h at room temperature to block non-specific cell adhesion. Prior to culturing cells, Pluronic F-127 solution was removed, and cell culture substrates were rinsed three times with PBS.

### 4.3. Western Blot, Active Rho GTPases Assay

Rabbit anti c-Jun antibody (E254) and rabbit anti Sox-2 antibody (ab97959) were purchased from Abcam (Cambridge, UK). Rabbit anti p75NTR antibody (8238), rabbit anti RhoA antibody (2117), rabbit anti Rac1 antibody (4651), mouse anti β-Actin antibody (3700), rabbit anti MKK7 antibody (4172), rabbit anti phospho-MKK7 antibody (4171), rabbit anti phosphor-JNK antibody (4668), rabbit anti JNK antibody (9252), rabbit anti Sox-2 antibody (3579), anti-rabbit IgG, HRP-linked Antibody (7074) anti-mouse IgG, HRP-linked Antibody (7076), and an active Rho detection kit (8820) were purchased from Cell Signaling Technology (Danvers, MA, USA). Human PAK-1 PBD protein (agarose free) (14-864) was purchased from Sigma-Aldrich. Mouse anti YAP/TAZ antibody (sc-101199) was purchased from Santa Cruz Biotechnology (Dallas, TX, USA). Alexa Flour 488 goat anti-mouse (A11001) and Alexa Flour 488 goat anti-rabbit (A11008) secondary were purchased from Thermo Fisher Scientific. Dithiothreitol (DTT) (97061-340) was purchased from VWR (Radnor, PA, USA).

For active Rho GTPase immunoprecipitation assays, cell lysates were prepared using a standard RIPA (radioimmunoprecipitation assay) buffer (ab156034, Abcam) lysis protocol [14]. Total protein levels of cell lysates were measured using a BCA protein assay (23225, Thermo Fisher) and quantified with a 96-well microplate reader (Bio-Rad, Hercules, CA, USA). Lysates containing equal amounts of protein (700 µg) were incubated with binding proteins (GST-Rhotekin-RBD for active Rho assay and human PAK-1 PBD for active Rac1 assay) and glutathione resin at 4 °C for 1 h with gentle rocking. Unbound and inactive Rho GTPases were separated by centrifugation at 6000× *g* for 30 s. Resin containing active Rho GTPases was washed using a washing buffer followed by centrifugation at 6000× *g* for 30 s three times before adding a reducing sample buffer (200 mM DTT in 2× SDS sample buffer) at room temperature for 2 min. The mixture was centrifuged at 6000× *g* for 30 min and the eluted sample was collected and heated at 96 °C for 5 min before proceeding to Western blot analysis.

For Western blot, samples were resolved on an 8–15% SDS-acrylamide gel. Proteins were transferred to a nitrocellulose membrane (10600004, GE Healthcare, Chicago, IL, USA) using a Trans-Blot Turbo system (Bio-Rad). Membranes were blocked with 4% BSA in TBST (Tris-buffered saline with 0.1% Tween-20) and incubated with the appropriate primary antibody overnight at 4 °C. Membranes were washed three times with TBST before incubating with HRP-linked secondary antibody for 1 h at room temperature. To develop blots, membranes were incubated with chemiluminescence (ECL) plus substrates (34577, Thermo Fisher) for 5 min at room temperature and imaged using a ChemiDoc MP imaging system (Bio-Rad). To quantify and normalize blots, images were analyzed with Image Lab software (Bio-Rad, v. 6.0.1).

### 4.4. siRNA Transfection

Rac1 siRNA (120600), siRNA negative control (4390843), Lipofectamine RNAiMAX transfection reagent (13778075), and Opti-MEM reduced serum medium (31985070) were purchased from Thermo Fisher Scientific. MKK7 siRNA (6323s) was purchased from Cell Signaling. To prepare SCs for siRNA transfection, 60,000 cells were seeded in a 60 mm Petri dish and incubated for 3 days. To prepare the siRNA–lipid complex, siRNA stock solution (10 µM) and Lipofectamine RNAiMAX were separately diluted with Opti-MEM reduced serum medium at 1:50 and 1:30 dilutions, respectively, before mixing together at a 1:1 volumetric ratio and incubating at room temperature for 5 min. The siRNA–lipid complex was added into normal SC culture medium to obtain a final concentration of 30 nM for Rac1 siRNA and 100 nM for MKK7 siRNA. SCs were treated with siRNA–lipid complex for 2 days before proceeding to quantitative analysis.

### 4.5. Immunofluorescent Staining and Cell Analysis

For immunofluorescence staining, SCs were fixed with 3.7% formaldehyde at room temperature for 15 min and permeabilized with 0.3% Triton X-100 for 5 min at 4 °C. Cells were then incubated with 3% BSA and 10% goat serum in PBS for 1 h at room temperature. Cells were incubated with either c-Jun (1:400), YAP/TAZ (1:500), or p75NTR (1:500) primary antibody overnight at 4 °C, followed by treatment with appropriate secondary antibody and rhodamine-phalloidin (R415, Thermo Fisher) for 1 h at room temperature. Cells were mounted to glass cover slides using antifade mounting solution with DAPI (P36971, Thermo Fisher) and sealed with nail polish. Coverslips were imaged using a Nikon Eclipse Ti2 inverted microscope with a Nikon DS-Qi2 camera.

Cellular properties were analyzed using Nikon NIS Elements software (v. 5.02.00). SC spreading area, nuclear elongation, and mean pixel fluorescent intensity of p75NTR were automatically detected and analyzed using Nikon analysis software. Mean pixel fluorescent intensities of c-Jun and Sox-2 were detected using the “ROI” (region of interest) and “Threshold” functions within Nikon NIS Elements software, as described elsewhere [17]. To measure and quantify YAP/TAZ activity, mean pixel YAP/TAZ fluorescent intensities of the nuclear area and the entire cellular area were measured by Nikon NIS Elements software. The ratio between nuclear and cytoplasmic mean pixel YAP/TAZ fluorescent intensity was calculated using Equation (1) (FITC: Fluorescein isothiocyanate):

(1) $$\begin{array}{l} \mathrm{AP}/\mathrm{TAZ}\;\mathrm{activity}=\frac{\mathrm{MeanFITC}\;\mathrm{of}\;\mathrm{nuclear}\;\mathrm{area}}{\mathrm{MeanFITC}\;\mathrm{of}\;\mathrm{cytoplasmic}\;\mathrm{area}}\\ =\frac{\mathrm{MeanFITC}\;\mathrm{of}\;\mathrm{nuclear}\;\mathrm{area}}{\left(\mathrm{Area}\;\mathrm{of}\;\mathrm{cell}\times \mathrm{MeanFITC}\;\mathrm{of}\;\mathrm{cell}-\mathrm{Area}\;\mathrm{of}\;\mathrm{nuclear}\times \mathrm{MeanFITC}\;\mathrm{of}\;\mathrm{nuclear}\;\mathrm{area}\right)/\mathrm{Area}\;\mathrm{of}\;\left(\mathrm{cell}-\mathrm{nuclear}\right)} \end{array}$$

### 4.6. Microarray Analysis

Total RNA of cultured SCs was isolated using an RNeasy Mini kit (74104, Qiagen, Hilden, Germany) according to the manufacturer’s instructions. RNA quality, including concentration and rRNA ratio (28s/18s), was checked with the Agilent 2100 Bioanalyzer using the RNA 6000 Nano Assay. Then, 100 ng of total RNA was used to generate biotin-labeled single-stranded complementary DNA (cDNA) using a GeneChip™ WT PLUS Reagent Kit (902280, Thermo Fisher). For single probe array, a hybridization cocktail containing 2.3 µg of fragmented cDNA was hybridized to Rat Clariom S Arrays (902934, Thermo Fisher). The array was incubated at 45 °C for 17 h in the Hybridization Oven 640 (Affymetrix) rotating at 60 rpm before washing and staining using the Fluidics Station 450 (Affymetrix) with the GeneChip™ Hybridization, Wash, and Stain kit (900720, Thermo Fisher). The array was scanned using the Affymetrix GeneChip Scanner 3000 7G and the .cel raw data file was generated by Command Console (Affymetrix).

Microarray data was imported to RStudio (https://rstudio.com/, accessed on 20 December 2020), for further analysis. Briefly, RMA normalization and log2 transformation were applied to the intensities of the probes across samples. Probes with a fold change greater than 1.5 or lower than −1.5 were considered when comparing SCs with different ECM stiffness, while probes with a fold change greater than 2 or lower than −2 were considered when comparing SCs with different spreading areas and different extents of elongation. Within RStudio, the dist function (method = “Euclidean”) was used to compute the distance between the rows of the data matrix, the hclust (method = “complete”) function was used to perform hierarchical clustering, and the heatmap.2 function was used to generate heatmaps that represent fold changes of critical genes. All microarray data are deposited in the NCBI Gene Expression Omnibus (GEO). The accession number is GSE165206.

### 4.7. Statistics

Origin Lab 9.1 and Excel were used to perform statistical analyses. One-way analysis of variance (ANOVA) followed by Tukey’s post hoc test or Student’s *t*-test was used to determine statistically significant differences between two independent experimental groups. *p* ≤ 0.05 was considered significant difference, * denotes *p* ≤ 0.05, ** denotes *p* ≤ 0.005, and *** denotes *p* ≤ 0.0005. All data are represented as mean values ± standard error of the mean (SEM).

## 5. Conclusions

SCs are highly plastic and regulated by extracellular mechanical cues such as ECM stiffness. The dynamic reciprocity between ECM and the cell leads to varying cell morphology, which greatly impacts the regenerative phenotype of SCs. We show that ECM stiffness and cell spreading inhibit SC regenerative markers by the activation of RhoA and YAP/TAZ signaling. Inhibition of RhoA and YAP/TAZ significantly promotes SC regenerative markers, with RhoA inhibition resulting in a higher expression of key markers. The upregulation of markers resulting from RhoA or YAP/TAZ inhibition requires activation of Rac1/MKK7/JNK. Silencing Rac1 significantly downregulates SC regenerative markers, even in the presence of RhoA and YAP/TAZ inhibitors. Rac1/MKK7/JNK signaling also plays a critical role in promoting SC regenerative markers in elongated SCs. SCs with the same spreading area but different extents of elongation show comparable activity of regenerative markers upon Rac1 or MKK7 inhibition. These findings show the complex interplay between the ECM and SCs in phenotype specification and should be considered in future nerve regeneration approaches.

## Data Availability

The microarray raw data can be accessed through GEO database, the accession number is GSE165206.

## Supplementary Materials

The following are available online at https://www.mdpi.com/article/10.3390/ijms22094821/s1.

## References

[1] Jessen K.R., Mirsky R. (2019). The Success and Failure of the Schwann Cell Response to Nerve Injury. Front. Cell. Neurosci..

[2] Nocera G., Jacob C. (2020). Mechanisms of Schwann cell plasticity involved in peripheral nerve repair after injury. Cell. Mol. Life Sci..

[3] Ibanez C.F., Simi A. (2012). p75 neurotrophin receptor signaling in nervous system injury and degeneration: Paradox and opportunity. Trends Neurosci..

[4] Roberts S.L., Dun X.-P., Doddrell R.D.S., Mindos T., Drake L.K., Onaitis M.W., Florio F., Quattrini A., Lloyd A.C., D’Antonio M. (2017). Sox2 expression in Schwann cells inhibits myelination in vivo and induces influx of macrophages to the nerve. Development.

[5] Deumens R., Bozkurt A., Meek M.F., Marcus M.A.E., Joosten E.A.J., Weis J., Brook G.A. (2010). Repairing injured peripheral nerves: Bridging the gap. Prog. Neurobiol..

[6] Parrinello S., Napoli I., Ribeiro S., Digby P.W., Fedorova M., Parkinson D.B., Doddrell R.D.S., Nakayama M., Adams R.H., Lloyd A.C. (2010). EphB signaling directs peripheral nerve regeneration through Sox2-dependent Schwann cell Sorting. Cell.

[7] Fu S.Y., Gordon T. (1997). The cellular and molecular basis of peripheral nerve regeneration. Mol. Neurobiol..

[8] Gordon T., Wood P., Sulaiman O.A.R. (2018). Long-Term Denervated Rat Schwann Cells Retain Their Capacity to Proliferate and to Myelinate Axons in vitro. Front. Cell. Neurosci..

[9] Frantz C., Stewart K.M., Weaver V.M. (2010). The extracellular matrix at a glance. J. Cell Sci..

[10] Belin S., Zuloaga K.L., Poitelon Y. (2017). Influence of Mechanical Stimuli on Schwann Cell Biology. Front. Cell. Neurosci..

[11] Harris G.M., Madigan N.N., Lancaster K.Z., Enquist L.W., Windebank A.J., Schwartz J., Schwarzbauer J.E. (2017). Nerve Guidance by a Decellularized Fibroblast Extracellular Matrix. Matrix Biol..

[12] Gu Y., Ji Y., Zhao Y., Liu Y., Ding F., Gu X., Yang Y. (2012). The influence of substrate stiffness on the behavior and functions of Schwann cells in culture. Biomaterials.

[13] Evans E.B., Brady S.W., Tripathi A., Hoffman-kim D. (2018). Schwann cell durotaxis can be guided by physiologically relevant stiffness gradients. Biomater. Res..

[14] Xu Z., Orkwis J.A., DeVine B.M., Harris G.M. (2019). Extracellular matrix cues modulate Schwann cell morphology, proliferation, and protein expression. J. Tissue Eng. Regen. Med..

[15] Coillard A., Segura E. (2019). In vivo Differentiation of Human Monocytes. Front. Immunol..

[16] Chernousov M.A., Yu W.-M., Chen Z.-L., Carey D.J., Strickland S. (2008). Regulation of Schwann cell function by the extracellular matrix. Glia.

[17] Xu Z., Orkwis J.A., Harris G.M. (2020). Preparation of Tunable Extracellular Matrix Microenvironments to Evaluate Schwann Cell Phenotype Specification. J. Vis. Exp..

[18] Chen C.S. (2008). Mechanotransduction—A field pulling together?. J. Cell Sci..

[19] Rausch V., Hansen C.G. (2020). The Hippo Pathway, YAP/TAZ, and the Plasma Membrane. Trends Cell Biol..

[20] Piccolo S., Dupont S., Cordenonsi M. (2014). The Biology of YAP/TAZ: Hippo Signaling and Beyond. Physiol. Rev..

[21] Urbanski M.M., Kingsbury L., Moussouros D., Kassim I., Mehjabeen S., Paknejad N., Melendez-Vasquez C.V. (2016). Myelinating glia differentiation is regulated by extracellular matrix elasticity. Sci. Rep..

[22] Grove M., Kim H., Santerre M., Krupka A.J., Han S.B., Zhai J., Cho J.Y., Park R., Harris M., Kim S. (2017). YAP/TAZ initiate and maintain schwann cell myelination. Elife.

[23] Sophie B., Jacob H., Jordan V.J.S., Yungki P., Laura F.M., Yannick P. (2019). YAP and TAZ Regulate Cc2d1b and Purbeta in Schwann Cells. Front. Mol. Neurosci..

[24] Grove M., Lee H., Zhao H., Son Y.J. (2020). Axon-dependent expression of YAP/TAZ mediates Schwann cell remyelination but not proliferation after nerve injury. Elife.

[25] Poitelon Y., Lopez-Anido C., Catignas K., Berti C., Palmisano M., Williamson C., Ameroso D., Abiko K., Hwang Y., Gregorieff A. (2016). YAP and TAZ control peripheral myelination and the expression of laminin receptors in Schwann cells. Nat. Neurosci..

[26] Lopez-Anido C., Poitelon Y., Gopinath C., Moran J.J., Ma K.H., Law W.D., Antonellis A., Feltri M.L., Svaren J. (2016). Tead1 regulates the expression of Peripheral Myelin Protein 22 during Schwann cell development. Hum. Mol. Genet..

[27] Mindos T., Dun X.P., North K., Doddrell R.D., Schulz A., Edwards P., Russell J., Gray B., Roberts S.L., Shivane A. (2017). Merlin controls the repair capacity of Schwann cells after injury by regulating Hippo/YAP activity. J. Cell Biol..

[28] Hall A. (1998). Rho GTPases and the Actin Cytoskeleton. Science.

[29] Provenzano P.P., Keely P.J. (2011). Mechanical signaling through the cytoskeleton regulates cell proliferation by coordinated focal adhesion and Rho GTPase signaling. J. Cell Sci..

[30] Etienne-manneville S.A.H. (2002). Rho GTPases in cell biology. Nature.

[31] O’Brien L.E., Jou T.-S., Pollack A.L., Zhang Q., Hansen S.H., Yurchenco P., Mostov K.E. (2001). Rac1 orientates epithelial apical polarity through effects on basolateral laminin assembly. Nat. Cell Biol..

[32] Chauhan B.K., Lou M., Zheng Y., Lang R.A. (2011). Balanced Rac1 and RhoA activities regulate cell shape and drive invagination morphogenesis in epithelia. Proc. Natl. Acad. Sci. USA.

[33] Melendez-Vasquez C.V., Einheber S., Salzer J.L. (2004). Rho kinase regulates schwann cell myelination and formation of associated axonal domains. J. Neurosci..

[34] Marinissen M.J., Chiariello M., Tanos T., Bernard O., Narumiya S., Gutkind J.S. (2004). The small GTP-binding protein RhoA regulates c-jun by a ROCK-JNK signaling axis. Mol. Cell.

[35] Nodari A., Zambroni D., Quattrini A., Court F.A., D’Urso A., Recchia A., Tybulewicz V.L., Wrabetz L., Feltri M.L. (2007). Beta1 integrin activates Rac1 in Schwann cells to generate radial lamellae during axonal sorting and myelination. J. Cell Biol..

[36] Park H.T., Feltri M.L. (2011). Rac1 GTPase controls myelination and demyelination. Bioarchitecture.

[37] Shin Y.K., Jang S.Y., Park J.Y., Park S.Y., Lee H.J., Suh D.J., Park H.T. (2013). The Neuregulin-Rac-MKK7 pathway regulates antagonistic c-jun/Krox20 expression in Schwann cell dedifferentiation. Glia.

[38] Martin E., Ouellette M.H., Jenna S. (2016). Rac1/RhoA antagonism defines cell-to-cell heterogeneity during epidermal morphogenesis in nematodes. J. Cell Biol..

[39] Bros M., Haas K., Moll L., Grabbe S. (2019). RhoA as a Key Regulator of Innate and Adaptive Immunity. Cells.

[40] Hong E.-H., Kim J.-Y., Kim J.-H., Lim D.-S., Kim M., Kim J.-Y. (2018). BIG2-ARF1-RhoA-mDia1 Signaling Regulates Dendritic Golgi Polarization in Hippocampal Neurons. Mol. Neurobiol..

[41] Liu H.W., Halayko A.J., Fernandes D.J., Harmon G.S., McCauley J.A., Kocieniewski P., McConville J., Fu Y., Forsythe S.M., Kogut P. (2003). The RhoA/Rho kinase pathway regulates nuclear localization of serum response factor. Am. J. Respir. Cell Mol. Biol..

[42] Koo J.H., Plouffe S.W., Meng Z., Lee D.H., Yang D., Lim D.S., Wang C.Y., Guan K.L. (2020). Induction of AP-1 by YAP/TAZ contributes to cell proliferation and organ growth. Genes Dev..

[43] Li Z., Razavi P., Li Q., Toy W., Liu B., Ping C., Hsieh W., Sanchez-Vega F., Brown D.N., Da Cruz Paula A.F. (2018). Loss of the FAT1 Tumor Suppressor Promotes Resistance to CDK4/6 Inhibitors via the Hippo Pathway. Cancer Cell.

[44] Schulze-Topphoff U., Prat A., Prozorovski T., Siffrin V., Paterka M., Herz J., Bendix I., Ifergan I., Schadock I., Mori M.A. (2009). Activation of kinin receptor B1 limits encephalitogenic T lymphocyte recruitment to the central nervous system. Nat. Med..

[45] Mburu P., Romero M.R., Hilton H., Parker A., Townsend S., Kikkawa Y., Brown S.D. (2010). Gelsolin plays a role in the actin polymerization complex of hair cell stereocilia. PLoS ONE.

[46] Boerboom A., Dion V., Chariot A., Franzen R. (2017). Molecular Mechanisms Involved in Schwann Cell Plasticity. Front. Mol. Neurosci..

[47] Dahlin L.B. (2014). Nerve injury-induced c-Jun activation in Schwann cells is JNK independent. BioMed Res. Int..

[48] Schmid D., Zeis T., Schaeren-Wiemers N. (2014). Transcriptional regulation induced by cAMP elevation in mouse Schwann cells. ASN Neuro.

[49] Wang H., Zhang P., Yu J., Zhang F., Dai W., Yi S. (2019). Matrix metalloproteinase 7 promoted Schwann cell migration and myelination after rat sciatic nerve injury. Mol. Brain.

[50] Jessen K.R., Arthur-Farraj P. (2019). Repair Schwann cell update: Adaptive reprogramming, EMT, and stemness in regenerating nerves. Glia.

[51] Li Y., Ye Z., Chen S., Pan Z., Zhou Q., Li Y.Z., Shuai W.D., Kuang C.M., Peng Q.H., Shi W. (2018). ARHGEF19 interacts with BRAF to activate MAPK signaling during the tumorigenesis of non-small cell lung cancer. Int. J. Cancer.

[52] Gawlak G., Tian Y., O’Donnell J.J., Tian X., Birukova A.A., Birukov K.G. (2014). Paxillin mediates stretch-induced Rho signaling and endothelial permeability via assembly of paxillin-p42/44MAPK-GEF-H1 complex. FASEB J..

[53] Cayuso J., Xu Q., Addison M., Wilkinson D.G. (2019). Actomyosin regulation by Eph receptor signaling couples boundary cell formation to border sharpness. Elife.

[54] Mullin B.H., Mamotte C., Prince R.L., Wilson S.G. (2014). Influence of ARHGEF3 and RHOA knockdown on ACTA2 and other genes in osteoblasts and osteoclasts. PLoS ONE.

[55] Joung I., Yoo M., Woo J.H., Chang C.Y., Heo H., Kwon Y.K. (2010). Secretion of EGF-like domain of heregulinbeta promotes axonal growth and functional recovery of injured sciatic nerve. Mol. Cells.

[56] Castelnovo L.F., Bonalume V., Melfi S., Ballabio M., Colleoni D., Magnaghi V. (2017). Schwann cell development, maturation and regeneration: A focus on classic and emerging intracellular signaling pathways. Neural Regen. Res..

[57] Sun Z., Huang S., Li Z., Meininger G.A. (2012). Zyxin is involved in regulation of mechanotransduction in arteriole smooth muscle cells. Front. Physiol..

[58] Sabra H., Brunner M., Mandati V., Wehrle-Haller B., Lallemand D., Ribba A.S., Chevalier G., Guardiola P., Block M.R., Bouvard D. (2017). beta1 integrin-dependent Rac/group I PAK signaling mediates YAP activation of Yes-associated protein 1 (YAP1) via NF2/merlin. J. Biol. Chem..

[59] Lopez-Fagundo C., Bar-Kochba E., Livi L.L., Hoffman-Kim D., Franck C. (2014). Three-dimensional traction forces of Schwann cells on compliant substrates. J. R. Soc. Interface.

[60] Zhang L., Yang X., Yue Y., Ye J., Yao Y., Fu Y., Li G., Yao Q., Lin Y., Gong P. (2015). Cyclic mechanical stress modulates neurotrophic and myelinating gene expression of Schwann cells. Cell Prolif..

[61] Zhou W., Stukel J.M., Cebull H.L., Willits R.K. (2016). Tuning the Mechanical Properties of Poly(Ethylene Glycol) Microgel-Based Scaffolds to Increase 3D Schwann Cell Proliferation. Macromol. Biosci..

[62] Rosso G., Guck J. (2019). Mechanical changes of peripheral nerve tissue microenvironment and their structural basis during development. APL Bioeng..

[63] Wells R.G. (2013). Tissue mechanics and fibrosis. Biochim. Biophys. Acta Mol. Basis Dis..

[64] Asano S., Ito S., Takahashi K., Furuya K., Kondo M., Sokabe M., Hasegawa Y. (2017). Matrix stiffness regulates migration of human lung fibroblasts. Physiol. Rep..

[65] Ozcelikkale A., Dutton J.C., Grinnell F., Han B. (2017). Effects of dynamic matrix remodelling on en masse migration of fibroblasts on collagen matrices. J. R. Soc. Interface.

[66] El-Mohri H., Wu Y., Mohanty S., Ghosh G. (2017). Impact of matrix stiffness on fibroblast function. Mater. Sci. Eng. C Mater. Biol. Appl..

[67] Blaker J.J., Faroni A., Li X., Gough J.E., Magaz A., Reid A.J. (2018). Bioactive Silk-Based Nerve Guidance Conduits for Augmenting Peripheral Nerve Repair. Adv. Healthc. Mater..

[68] Ahmed M., Ffrench-Constant C. (2016). Extracellular Matrix Regulation of Stem Cell Behavior. Curr. Stem Cell Rep..

[69] Chang T.Y., Chen C., Lee M., Chang Y.C., Lu C.H., Lu S.T., Wang D.Y., Wang A., Guo C.L., Cheng P.L. (2017). Paxillin facilitates timely neurite initiation on soft-substrate environments by interacting with the endocytic machinery. Elife.

[70] Kovar H., Bierbaumer L., Radic-Sarikas B. (2020). The YAP/TAZ Pathway in Osteogenesis and Bone Sarcoma Pathogenesis. Cells.

[71] Boopathy G.T.K., Hong W. (2019). Role of Hippo Pathway-YAP/TAZ Signaling in Angiogenesis. Front. Cell Dev. Biol..

[72] Jessen K.R., Mirsky R. (2016). The repair Schwann cell and its function in regenerating nerves. J. Physiol..

[73] Aragona M., Panciera T., Manfrin A., Giulitti S., Michielin F., Elvassore N., Dupont S., Piccolo S. (2013). A mechanical checkpoint controls multicellular growth through YAP/TAZ regulation by actin-processing factors. Cell.

[74] Deng Y., Wu L.M.N., Bai S., Zhao C., Wang H., Wang J., Xu L., Sakabe M., Zhou W., Xin M. (2017). A reciprocal regulatory loop between TAZ/YAP and G-protein Galphas regulates Schwann cell proliferation and myelination. Nat. Commun..

[75] Tan D., Wen J., Li L., Wang X., Qian C., Pan M., Lai M., Deng J., Hu X., Zhang H. (2018). Inhibition of RhoA-Subfamily GTPases Suppresses Schwann Cell Proliferation Through Regulating AKT Pathway Rather Than ROCK Pathway. Front. Cell. Neurosci..

[76] Yamauchi J., Chan J.R., Shooter E.M. (2004). Neurotrophins regulate Schwann cell migration by activating divergent signaling pathways dependent on Rho GTPases. Proc. Natl. Acad. Sci. USA.

[77] Mantuano E., Jo M., Gonias S.L., Campana W.M. (2010). Low density lipoprotein receptor-related protein (LRP1) regulates Rac1 and RhoA reciprocally to control Schwann cell adhesion and migration. J. Biol. Chem..

[78] Arthur W.T., Burridge K., Carolina N., Hill C., Carolina N. (2001). RhoA Inactivation by p190RhoGAP Regulates Cell Spreading and Migration by Promoting Membrane Protrusion and Polarity. Mol. Biol. Cell.

[79] Colciago A., Melfi S., Giannotti G., Bonalume V., Ballabio M., Caffino L., Fumagalli F., Magnaghi V. (2015). Tumor suppressor Nf2/merlin drives Schwann cell changes following electromagnetic field exposure through Hippo-dependent mechanisms. Cell Death Discov..

[80] Benninger Y., Thurnherr T., Pereira J.A., Krause S., Wu X., Chrostek-Grashoff A., Herzog D., Nave K.A., Franklin R.J., Meijer D. (2007). Essential and distinct roles for cdc42 and rac1 in the regulation of Schwann cell biology during peripheral nervous system development. J. Cell Biol..

[81] Sanz-moreno V., Gadea G., Ahn J., Paterson H., Marra P., Pinner S., Sahai E., Marshall C.J. (2008). Rac Activation and Inactivation Control Plasticity of Tumor Cell Movement. Cell.

[82] Dupont S., Morsut L., Aragona M., Enzo E., Giulitti S., Cordenonsi M., Zanconato F., Le Digabel J., Forcato M., Bicciato S. (2011). Role of YAP/TAZ in mechanotransduction. Nature.

[83] Doddrell R.D., Dun X.P., Shivane A., Feltri M.L., Wrabetz L., Wegner M., Sock E., Hanemann C.O., Parkinson D.B. (2013). Loss of SOX10 function contributes to the phenotype of human Merlin-null schwannoma cells. Brain.

[84] Ahmad I., Fernando A., Gurgel R., Jason Clark J., Xu L., Hansen M.R. (2015). Merlin status regulates p75(NTR) expression and apoptotic signaling in Schwann cells following nerve injury. Neurobiol. Dis..

[85] Shaw R.J., Paez J.G., Curto M., Yaktine A., Pruitt W.M., Saotome I., O’Bryan J.P., Gupta V., Ratner N., Der C.J. (2001). The Nf2 Tumor Suppressor, Merlin, Functions in Rac-Dependent Signaling. Dev. Cell.

[86] Yamauchi J., Miyamoto Y., Chan J.R., Tanoue A. (2008). ErbB2 directly activates the exchange factor Dock7 to promote Schwann cell migration. J. Cell Biol..

[87] Yamauchi J., Chan J.R., Shooter E.M. (2003). Neurotrophin 3 activation of TrkC induces Schwann cell migration through the c-Jun N-terminal kinase pathway. Proc. Natl. Acad. Sci. USA.

[88] Manchanda P.K., Jones G.N., Lee A.A., Pringle D.R., Zhang M., Yu L., La Perle K.M., Kirschner L.S. (2013). Rac1 is required for Prkar1a-mediated Nf2 suppression in Schwann cell tumors. Oncogene.

[89] Zhang X., Zhao H., Li Y., Xia D., Yang L., Ma Y., Li H. (2018). The role of YAP/TAZ activity in cancer metabolic reprogramming. Mol. Cancer.

[90] Ju M.S., Lin C.C.K., Chang C.T. (2017). Researches on biomechanical properties and models of peripheral nerves—A review. J. Biomech. Sci. Eng..

[91] Cady E., Orkwis J.A., Weaver R., Conlin L., Madigan N.N., Harris G.M. (2020). Micropatterning Decellularized ECM as a Bioactive Surface to Guide Cell Alignment, Proliferation, and Migration. Bioengineering.

