# Supplementary material for: Cell Shape and Matrix Stiffness Impact Schwann Cell Plasticity via YAP/TAZ and Rho GTPases
Source: Int J Mol Sci. 2021 May 1;22(9):4821. doi: 10.3390/ijms22094821 (PMC8124465; doi:10.3390/ijms22094821)
Supplement: Supplementary file 1 [file ijms-22-04821-s001.zip › ijms-1182787-supplementary.pdf]

Cell Shape and Matrix Stiffness Impact Schwann Cell Plasticity via YAP/TAZ and RhoGTPases

Zhenyuan Xu[a], Jacob A. Orkwis[a], and Greg M. Harris[a,b,c]

[a]Department of Chemical and Environmental Engineering, University of Cincinnati, Cincinnati, OH 45221

[b]Department of Biomedical Engineering, University of Cincinnati, Cincinnati, OH 45221

[c]Neuroscience Graduate Program, University of Cincinnati College of Medicine, Cincinnati, OH 45267

Supplemental Information

---

* Corresponding author

Greg Harris, Ph.D.

Department of Chemical & Environmental Engineering

University of Cincinnati

2901 Woodside Drive

Cincinnati, OH 45221

Ph: (513) 556-4167

Fax: (513) 556-3473

Email: gregory.harris@uc.edu

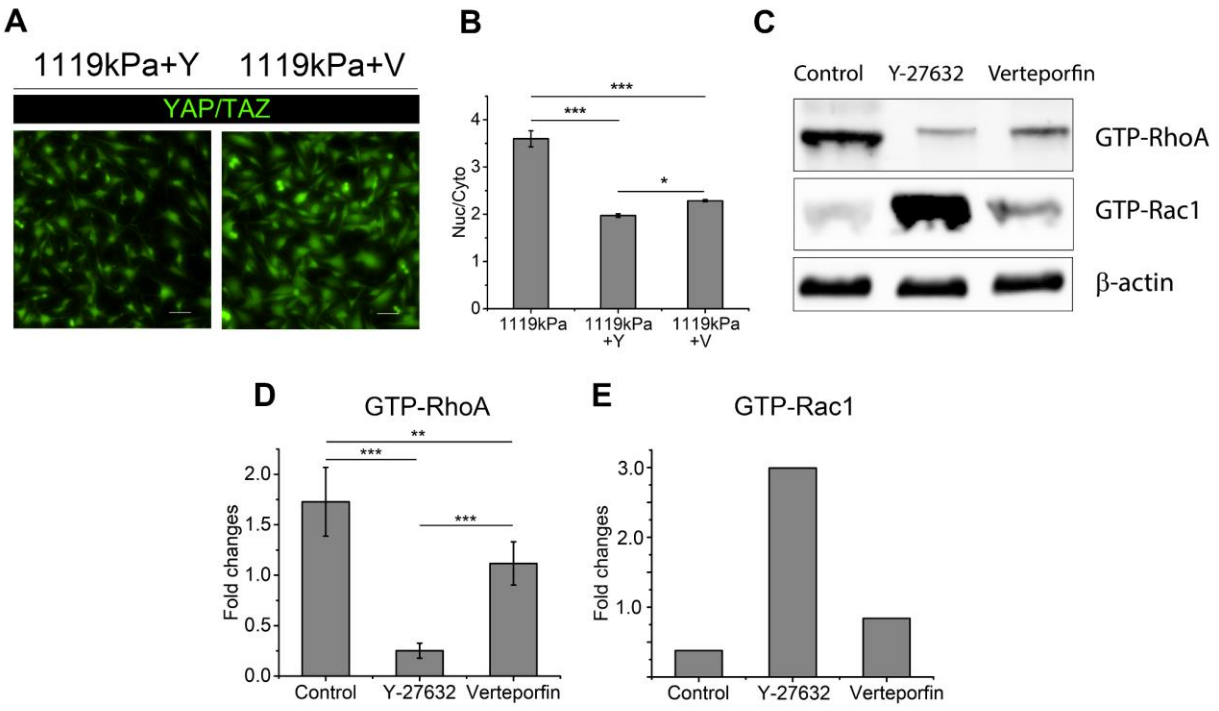

Figure S1: (A-B) Nuclear (active) YAP/TAZ is inhibited by Y-27632 (ROCK) and Verteporfin (YAP/TAZ). For YAP/TAZ fluorescent intensity quantification, n = 3 unique trials with a minimum of 35 cells were quantified per trial. Scale bar = 50 um. (C-E) When RhoA and YAP/TAZ are inhibited, active RhoA is downregulated while active Rac1 is upregulated. For Western blot analysis, 3 separate lysates were created to quantify GTP-RhoA and 1 lysate to quantify GTP-Rac1. Data is presented as mean ± SEM. *p<0.05, **p<0.005, ***p<0.0005.

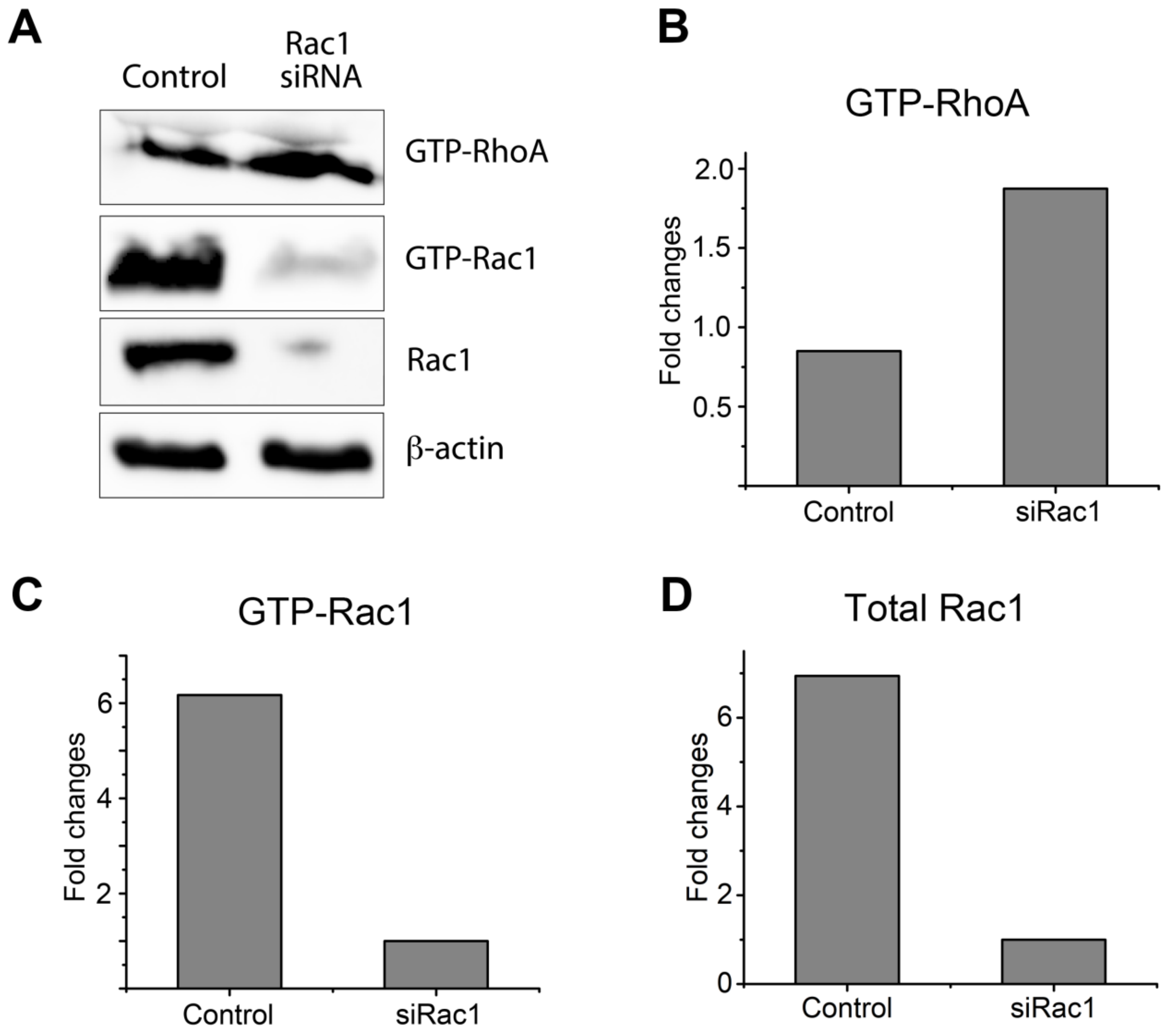

Figure S2: (A-D) siRac1 transfection significantly downregulates Rac1 activity while increasing RhoA activity.

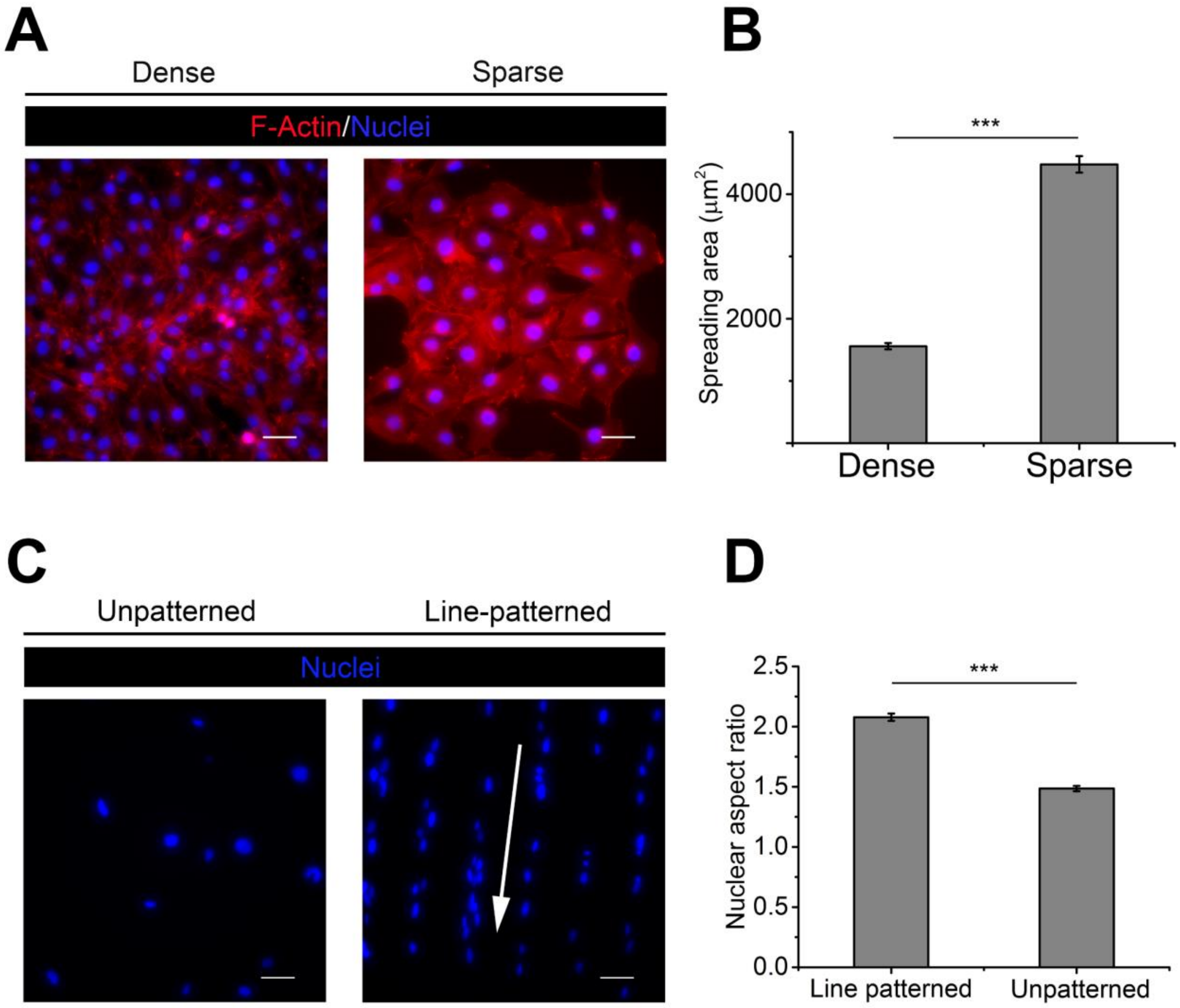

Figure S3: (A-B) F-actin (red) showing the average cell spreading area was controlled by varying initial cell seeding density. 3 unique trials with a minimum of 74 cells per trial were quantified. Scale bar = 50 um. (C-D) Nuclear (blue) elongation was promoted using line-patterned substrates when compared to cells on unpatterned surfaces. 6 unique trials with a minimum of 41 cells per trial were quantified. Scale bar = 50 um. White arrows indicate the direction of the line patterns. Data is presented as mean ± SEM. *p<0.05, **p<0.005, ***p<0.0005.

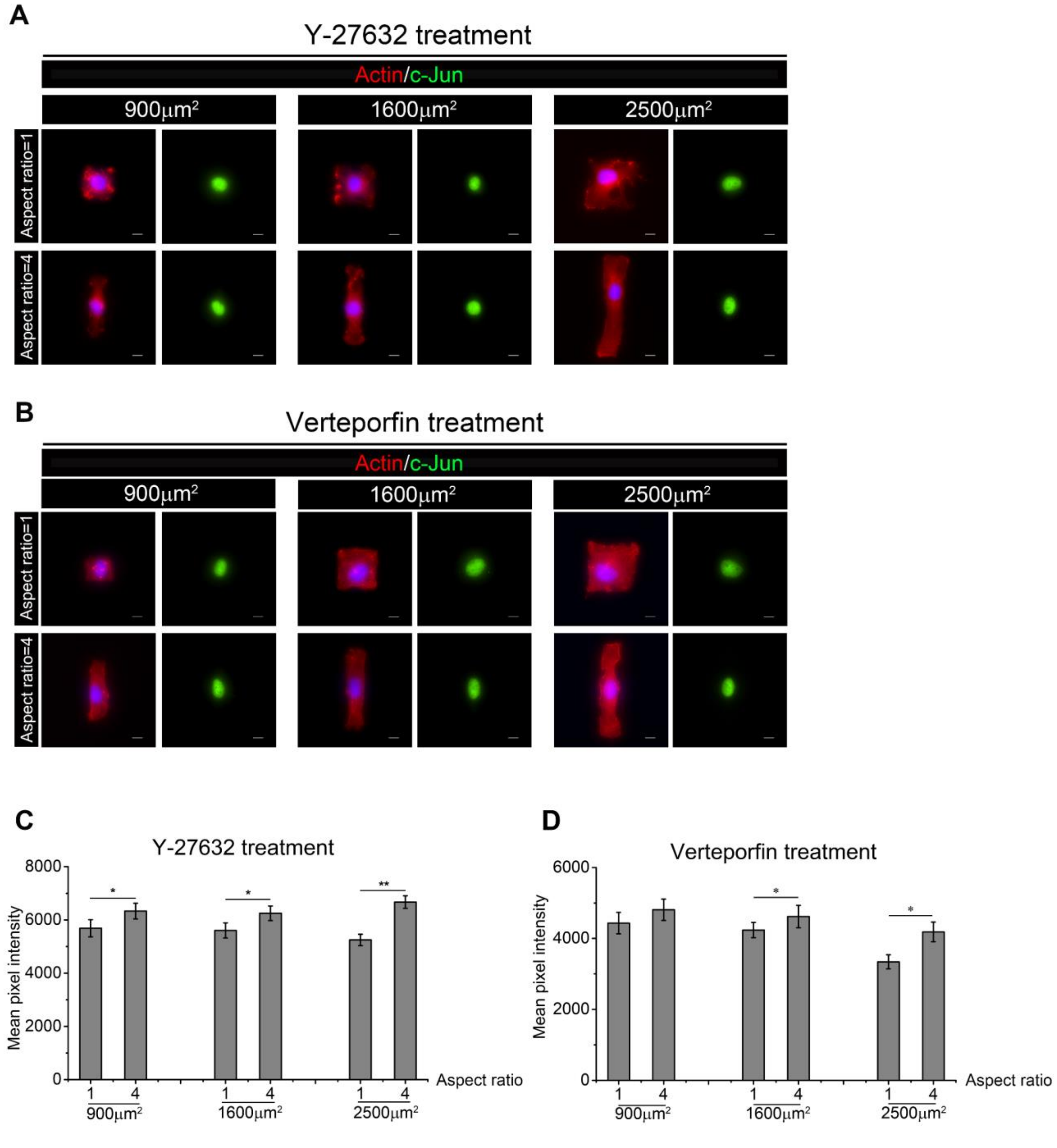

Figure S4: SCs on micropatterned, cell adhesive islands with variable spreading area and elongation (A) without treatment and (B) with Y-27632 and verteporfin treatment. SCs are visualized for nucleus (blue), F-actin (red), and c-Jun (green). Scale bar: = 10 um (C-D) The mean pixel intensity of c-Jun was quantified to show differences in expression across cell area and cell elongation with Y-27632 or verteporfin treatment. 5 unique trials with a minimum of 51 single micropatterned SCs per trial were quantified. Data is presented as mean ± SEM. *p<0.05, **p<0.005, ***p<0.0005.

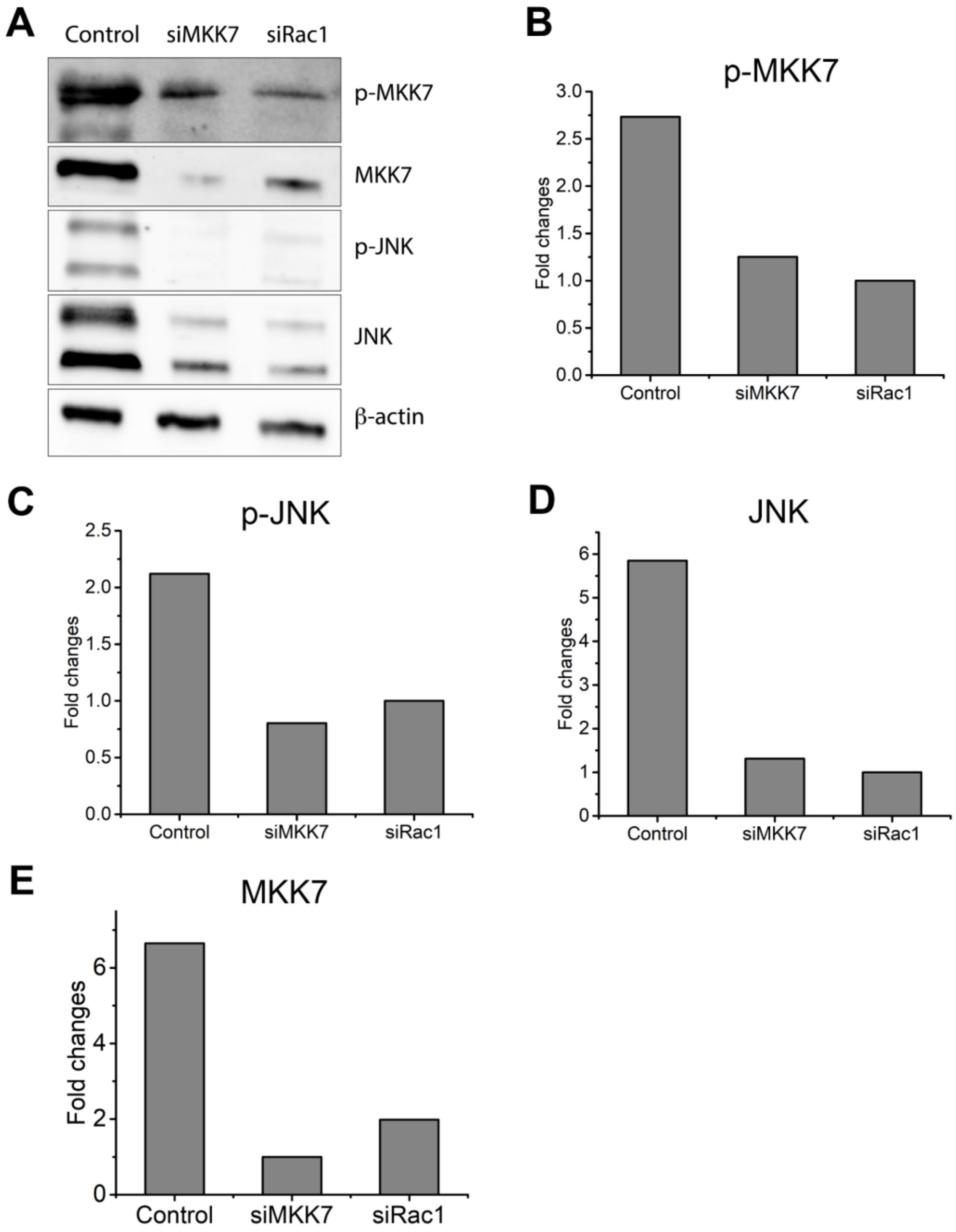

Figure S5: (A-D) Western blot analysis of phosphorylated-MKK7, phosphorylated-JNK, and total JNK upon siMKK7 and siRac1 transfection.

Table S1

		Conditions		p value
Western Blot	GTP-RhoA	1119 kPa	8.67 kPa	0.001941747
	GTP Rac1	1119 kPa	8.67 kPa	0.038639998
	c-Jun	1119 kPa	8.67 kPa	0.024560145
	p75NTR	1119 kPa	8.67 kPa	0.017519102
Staining	YAP/TAZ	1119 kPa	8.67 kPa	0
Western Blot	c-Jun	Control 8.67	Control 1119	0.00333
		Y 1119	Control 1119	0
		Y 1119	Control 8.67	0
		Y 8.67	Control 1119	0
		Y 8.67	Control 8.67	0
		Y 8.67	Y 1119	0.00676614
		V 1119	Control 1119	0.0007288
		V 1119	Control 8.67	0.0008781
		V 1119	Y 1119	1.92738E-07
		V 1119	Y 8.67	0
		V 8.67	Control 1119	3.0885E-08
		V 8.67	Control 8.67	0.000509
		V 8.67	Y 1119	2.97037E-08
		V 8.67	Y 8.67	7.55059E-06
		V 8.67	V 1119	4.70563E-06
Western Blot	p75NTR	Control 8.67	Control 1119	0.007490434
		Y 1119	Control 1119	0
		Y 1119	Control 8.67	0
		Y 8.67	Control 1119	0
		Y 8.67	Control 8.67	0
		Y 8.67	Y 1119	0.116116524
		V 1119	Control 1119	0.053338261
		V 1119	Control 8.67	0.056898804
		V 1119	Y 1119	0.001990975
		V 1119	Y 8.67	0.001526881
		V 8.67	Control 1119	0.14230723
		V 8.67	Control 8.67	0.19657615
		V 8.67	Y 1119	0.00293879
		V 8.67	Y 8.67	0.002895325
		V 8.67	V 1119	0.001885195

Table S2

		Conditions		p value
Western Blot	c-Jun	Y+Empty	Y+siRac1	0.001110743
		V+Empty	V+siRac1	0.001775254
		Empty	siRac1	0.003378035
		Empty	V+siRac1	0.003868486
Western Blot	p75NTR	Y+Empty	Y+siRac1	0.000139718
		V+Empty	V+siRac1	0.00305854
		Empty	siRac1	0.005522349
		Empty	V+siRac1	0.000255004
Staining	p75NTR	Y	Control	0
		V	Control	0.001
		V	Y	4.01E-03
		Control+siRac1	Control	0.1005
		Control+siRac1	Y	0
		Control+siRac1	V	5.40E-09
		Y+siRac1	Control	0.15641
		Y+siRac1	Y	0
		Y+siRac1	V	2.00E-08
		Y+siRac1	Control+siRac1	1
		V+siRac1	Control	0.1616
		V+siRac1	Y	0
		V+siRac1	V	2.60E-08
		V+siRac1	Control+siRac1	1
		V+siRac1	Y+siRac1	1
Staining	Sox-2	Y	Control	8.03E-09
		V	Control	7.19E-05
		V	Y	8.12E-07
		Control+siRac1	Control	2.55E-08
		Control+siRac1	Y	0
		Control+siRac1	V	4.18E-09
		Y+siRac1	Control	1.77E-08
		Y+siRac1	Y	0
		Y+siRac1	V	2.78E-09
		Y+siRac1	Control+siRac1	0.9999
		V+siRac1	Control	5.41E-08
		V+siRac1	Y	0
		V+siRac1	V	3.93E-09
		V+siRac1	Control+siRac1	0.9925
		V+siRac1	Y+siRac1	0.99909

Table S3

3		Conditions		p value
Western Blot	GTP-RhoA	Sparse	Dense	0.016210457
	c-Jun	Sparse	Dense	0.013122015
	GTP-Rac1	Sparse	Dense	0.023649123
	p75NTR	Sparse	Dense	0.00076223
Western Blot	GTP-RhoA	Unpatterned	Line-patterned	0.136001196
	c-Jun	Unpatterned	Line-patterned	0.001248187
	GTP-Rac1	Unpatterned	Line-patterned	0.000975385
	p75NTR	Unpatterned	Line-patterned	0.00080756
Staining	YAP/TAZ	900 4:1	900 1:1	0.99968
		1600 1:1	900 1:1	0.40664
		1600 1:1	900 4:1	0.71501
		1600 4:1	900 1:1	0.422
		1600 4:1	900 4:1	0.71563
		1600 4:1	1600 1:1	1
		2500 1:1	900 1:1	0.000338
		2500 1:1	900 4:1	0.02752
		2500 1:1	1600 1:1	0.0077539
		2500 1:1	1600 4:1	0.83776
		2500 4:1	900 1:1	0.00031908
		2500 4:1	900 4:1	1.25474E-06
		2500 4:1	1600 1:1	0.48494
		2500 4:1	1600 4:1	0.000806611
		2500 4:1	2500 1:1	0.99943

Table S4

4		Conditions		p value
Staining	c-Jun	900-1 Y	900-1 Con	6.29E-05
		900-1 V	900-1 Con	0.92292
		900-1 V	900-1 Y	0.04283
		1600-1 Con	900-1 Con	0.8213
		1600-1 Con	900-1 Y	1.15E-07
		1600-1 Con	900-1 V	0.16207
		1600-1 Y	900-1 Con	2.07E-05
		1600-1 Y	900-1 Y	1
		1600-1 Y	900-1 V	0.07922
		1600-1 Y	1600-1 Con	2.60E-08
		1600-1 V	900-1 Con	0.9947
		1600-1 V	900-1 Y	0.00318
		1600-1 V	900-1 V	0.99986
		1600-1 V	1600-1 Con	0.0042259
		1600-1 V	1600-1 Y	0.00196
		2500-1 Con	900-1 Con	0.003575
		2500-1 Con	900-1 Y	3.04E-08
		2500-1 Con	900-1 V	0.00277
		2500-1 Con	1600-1 Con	0.0092224
		2500-1 Con	1600-1 Y	2.82E-08
		2500-1 Con	1600-1 V	0.0061
		2500-1 Y	900-1 Con	0.00737
		2500-1 Y	900-1 Y	0.96333
		2500-1 Y	900-1 V	0.4751
		2500-1 Y	1600-1 Con	2.93E-05
		2500-1 Y	1600-1 Y	0.009824
		2500-1 Y	1600-1 V	0.11887
		2500-1 Y	2500-1 Con	2.16E-08
		2500-1 V	900-1 Con	0.91699
		2500-1 V	900-1 Y	6.41E-05
		2500-1 V	900-1 V	0.3792
		2500-1 V	1600-1 Con	1
		2500-1 V	1600-1 Y	4.55E-05
		2500-1 V	1600-1 V	0.0059078
		2500-1 V	2500-1 Con	0.99797
		2500-1 V	2500-1 Y	0.0251
Staining	p75NTR	1600 1 C	900 1 C	0.96637
		2500 1 C	900 1 C	0.000061834
		2500 1 C	1600 1 C	0.00732175
		900 1 Y	900 1 C	0.027521241

		900 1 Y	1600 1 C	0.25442
		900 1 Y	2500 1 C	0.05647
		1600 1 Y	900 1 C	0.99608
		1600 1 Y	1600 1 C	0.0034485
		1600 1 Y	2500 1 C	0.2362
		1600 1 Y	900 1 Y	0.99999
		2500 1 Y	900 1 C	0.99799
		2500 1 Y	1600 1 C	0.99998
		2500 1 Y	2500 1 C	0.007590934
		2500 1 Y	900 1 Y	0.0049004
		2500 1 Y	1600 1 Y	0.0085619
		900 1 V	900 1 C	0.99995
		900 1 V	1600 1 C	0.99876
		900 1 V	2500 1 C	0.8607
		900 1 V	900 1 Y	0.0068563
		900 1 V	1600 1 Y	0.9486
		900 1 V	2500 1 Y	1
		1600 1 V	900 1 C	0.19283
		1600 1 V	1600 1 C	0.8849
		1600 1 V	2500 1 C	0.99992
		1600 1 V	900 1 Y	0.00357
		1600 1 V	1600 1 Y	0.04185
		1600 1 V	2500 1 Y	0.65512
		1600 1 V	900 1 V	0.44244
		2500 1 V	900 1 C	0.0397
		2500 1 V	1600 1 C	0.33706
		2500 1 V	2500 1 C	0.83972
		2500 1 V	900 1 Y	0.00126
		2500 1 V	1600 1 Y	0.00875
		2500 1 V	2500 1 Y	0.017896
		2500 1 V	900 1 V	0.0010167
		2500 1 V	1600 1 V	0.0395489

Table S5

5		Conditions		p value
Western Blot	p-MKK7	Line-patterned	Unpatterned	0.002722371
	p-JNK	Line-patterned	Unpatterned	0.003487516
	JNK	Line-patterned	Unpatterned	0.009162065
siMKK7	c-Jun	AR=1	AR=4	0.211790246
	Sox-2	AR=1	AR=4	0.264193035
	p75NTR	AR=1	AR=4	0.598926393
siRac1	c-Jun	AR=1	AR=4	0.497195542
	Sox-2	AR=1	AR=4	0.409719997
	p75NTR	AR=1	AR=4	0.509865251
